# A quantum chemical interaction energy dataset for accurately modeling protein-ligand interactions

**DOI:** 10.1038/s41597-023-02443-1

**Published:** 2023-09-12

**Authors:** Steven A. Spronk, Zachary L. Glick, Derek P. Metcalf, C. David Sherrill, Daniel L. Cheney

**Affiliations:** 1 Molecular Structure and Design, Bristol Myers Squibb Company, P. O. Box 5400, Princeton, NJ 08543 USA; 2 Center for Computational Molecular Science and Technology, School of Chemistry and Biochemistry, and School of Computational Science and Engineering, Georgia Institute of Technology, Atlanta, GA 30332-0400 USA

**Keywords:** Computational chemistry, Method development

## Abstract

Fast and accurate calculation of intermolecular interaction energies is desirable for understanding many chemical and biological processes, including the binding of small molecules to proteins. The Splinter [“Symmetry-adapted perturbation theory (SAPT0) protein-ligand interaction”] dataset has been created to facilitate the development and improvement of methods for performing such calculations. Molecular fragments representing commonly found substructures in proteins and small-molecule ligands were paired into >9000 unique dimers, assembled into numerous configurations using an approach designed to adequately cover the breadth of the dimers’ potential energy surfaces while enhancing sampling in favorable regions. ~1.5 million configurations of these dimers were randomly generated, and a structurally diverse subset of these were minimized to obtain an additional ~80 thousand local and global minima. For all >1.6 million configurations, SAPT0 calculations were performed with two basis sets to complete the dataset. It is expected that Splinter will be a useful benchmark dataset for training and testing various methods for the calculation of intermolecular interaction energies.

## Background & Summary

Noncovalent interactions (NCIs) are fundamental for nearly all chemical and biochemical phenomena, so there is a great need for accurate and accessible approaches for their characterization. Consequently, various tools and methods have been developed to calculate binding energies of noncovalent dimers, usually involving a trade-off between accuracy and resources required.

It is generally accepted that quantum mechanical (QM) methods provide the most accurate NCI energy calculations, but they tend to be computationally intensive, with time and memory demands that limit the size of systems that can be studied in practice to several hundred atoms. One commonly used QM method that balances reasonable accuracy with modest computational cost is zeroth-order symmetry-adapted perturbation theory (SAPT0), which has the added advantage of providing not just an NCI’s total interaction energy, but also its decomposition into physically meaningful contributions from electrostatics, exchange-repulsion, polarization/induction, and London dispersion effects^1^.

NCI energies can also be approximated using molecular mechanics (MM) or machine learning (ML) approaches. These methods require significant initial effort to develop an MM force field (FF) or ML model, usually involving many high-level QM calculations, but once such an FF or ML model is obtained, the calculations for new systems are much less computationally intensive. Consequently, they are much faster than QM and also can be applied to much larger systems, such as biological systems like protein-ligand complexes consisting of thousands of atoms. These advantages generally come at the expense of accuracy, because FFs and ML models are necessarily only approximations to the underlying QM data on which they are based. That said, advances in both FF development as well as ML continue to improve their accuracy^2–4^. In particular, ML models trained on large sets of QM data have proven to reasonably approximate QM-derived potential energy surfaces for covalent systems (e. g., relative energies of conformers of single molecules)^5,6^ and also NCIs^7–11^.

With the aim of developing fast and accurate methods for predicting SAPT0 total and component electrostatic, exchange, induction, and dispersion energies of protein-ligand complexes, we have created the Splinter (“SAPT0 protein-ligand interaction”) dataset. Splinter consists of ~1.7 million different configurations of >9000 unique molecular dimers involving 332 distinct small molecules that represent commonly found substructures in proteins and small-molecule ligands, all of which were used for SAPT0 energy calculations with two different basis sets. ~1.5M configurations were generated randomly from a nonuniform probability distribution from each dimer’s potential energy surface, tuned to broadly capture the intermolecular radial and angular dependence of the energy. Additionally, ~80K minimized structures were obtained by optimization of a diverse collection of these random configurations, which were included in the dataset “as is” and also with slight random perturbations as described below, accounting for the remaining ~160K configurations. This blended strategy allowed for adequate sampling of the complete potential energy surfaces, including very unfavorable regions (e. g., with severe van der Waals clash or electrostatic repulsion) and non-interacting regions (e. g., widely separated monomers), while ensuring that the shape and depth of the energy wells were accurately determined.

The primary motivation for Splinter was to build on our previous work involving AP-Net, an ML model that reproduces SAPT0 calculations of hydrogen-bonded complexes of the peptide bond mimetic N-methylacetamide^7^, by training and testing a more sophisticated and accurate version that is extended to a broader array of protein-ligand interactions. (A manuscript describing the new AP-Net model, based upon Splinter, will be published separately.) Splinter is uniquely suited for this purpose; although several datasets of QM-calculated NCI energies have been published^12–20^, to our knowledge, none are as extensive in size and chemical diversity, especially those that contain SAPT decomposition of the total energies. While ML model creation was the primary aim, Splinter is suitable for other applications as well, including the generation of more accurate force fields, especially those taking advantage of SAPT energy decomposition^21,22^, and the assessment of computationally cheaper QM methods for NCI prediction^23^. We expect that other researchers will find the Splinter dataset useful in the continuing development of methods for predicting NCI energies.

## Methods

### Monomer optimization

25 fragment-like molecules (17 neutral, 4 cationic, and 4 anionic) representing chemical moieties commonly found in proteins, both backbone and side chains, as well as four other species often associated with proteins (water and Na^+^, Cl^−^, and SO_4_^2−^ ions) comprised the “protein set.” 303 molecules (248 neutral, 23 cationic, and 32 anionic, including Na^+^ and Cl^−^ ions) representing functional groups commonly found in or associated with small molecule drug-like compounds comprised the “ligand set.” Both sets of fragments were selected in consultation with several medicinal chemists with significant experience in industrial drug discovery research. This information is summarized in Table 1, and the chemical structures of all molecules in the protein and ligand sets are shown in Supplementary Figs. S1 and S2, respectively.

**Table 1:** Types and counts of molecules, monomers, and interaction sites, broken down by charged state (neutral/cationic/anionic).

Entity	Protein set count	Ligand set count
Unique molecule	29 (18/5/6)	303 (248/23/32)
Monomer*	31 (19/5/7)	306 (250/23/33)
General interaction site	33 (21/5/7)	306 (250/23/33)
Hydrogen bond donor site	27 (14/12/1)	177 (143/32/2)
Hydrogen bond acceptor site	23 (11/1/11)	359 (293/0/66)
Lewis base site	23 (10/1/12)	338 (254/0/84)
Lewis acid site	10 (8/1/1)	138 (129/0/9)

*The term “monomer” includes distinct molecules and distinct conformers.

Each molecule/ion was provided in Simplified Molecular Input Line Entry System (SMILES) format to the OEChem (version 2019.Oct.2)^24^ and Omega Toolkit (version 2019.Oct.2)^25^ Python APIs for the generation of 3D molecular coordinates. Geometry optimizations were performed with Psi4 (version 1.3)^26^ using method B3LYP^27^ and a basis set appropriate for the charge of the monomer (cc-pVDZ for neutral and cationic, aug-cc-pVDZ for anionic)^28,29^. In most cases, the single minimum obtained for each monomer was used in dimer formation. In five exceptions (detailed below), it was determined that two conformers were different enough and sufficiently low in energy to warrant inclusion of both, yielding a total number of conformers in the protein and ligand sets of 31 and 306, respectively. Hereafter, the term “monomer” includes all of these: different molecules as well as distinct conformers of the same molecule. Monomer structures are available in the Splinter data repository^30^.

**Fig. 1:**
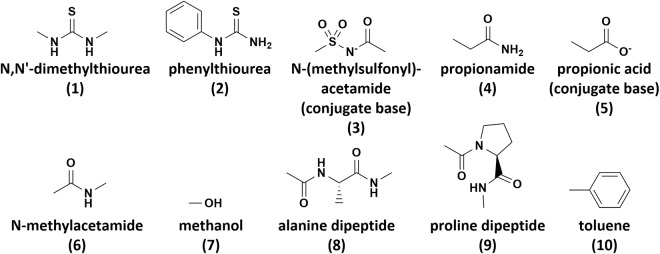
Specific molecular fragments discussed in the text.

The exceptions include three molecules in the ligand set: two thiourea molecules (**1** and **2** in Fig. 1), where the conformers differed by cis/trans isomerization of the larger substituent, and the sulfonylacetamide conjugate base (**3** in Fig. 1), where the conformers differed by rotation of the S-N bond to be either gauche or trans with respect to the sulfonyl methyl group. The other two exceptions were from the protein set. The propionamide and propionate fragments (**4** and **5** in Fig. 1, respectively) optimized to conformations where the terminal methyl lay in the amide/acid plane adjacent to the carbonyl O. However, there is known to be considerable variation in the orientation of the amide/carboxylate group in Asp/Asn/Glu/Gln side chains in protein structures^31^. To capture an important alternate conformation, one additional structure was obtained for each of these two fragments by rotating the O-C(carbonyl)-CH_2_-CH_3_ dihedral angle to 90°, retaining all other bond lengths, angles, and dihedral angles of their optimized conformations.

### Interaction site definition

**Fig. 2:**
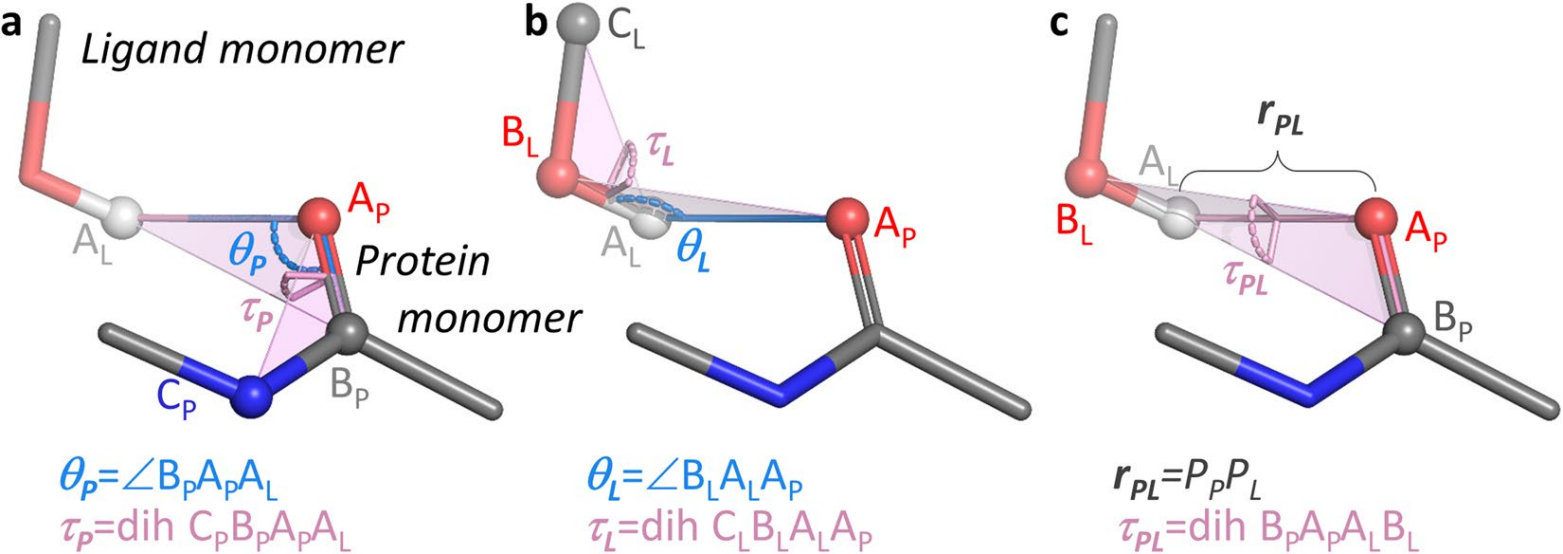
Intermolecular coordinates defining the configuration for a prototypical dimer. For the carbonyl HBA site of N-methylacetamide (protein set monomer; compound **6** in Fig. 1) and the hydroxyl HBD site of methanol (ligand set monomer; compound **7** in Fig. 1), points A, B, and C (with subscripts P and L to designate them as protein and ligand site points, respectively) were defined to emphasize the hydrogen-bond interaction. (**a**) The points and angular coordinate definitions associated with the N-methylacetamide HBA site. (**b**) The points and angular coordinate definitions associated with the methanol HBD site. (**c**) The coordinates involving both monomers equally.

Six coordinates are required to completely describe the intermolecular geometry between two interacting rigid monomers; these coordinates may be defined as one distance, two angles, and three dihedral angles involving three points from each monomer (Fig. 2). Consequently, sets of three noncollinear points (which did not necessarily coincide with atomic centers) were defined as “interaction sites” for all monomers. The primary point A of each site was centered on the atom or group of atoms making the most significant contact in a given interaction (A_P_ and A_L_ in Fig. 2; the subscripts P and L designate protein and ligand interaction site points, respectively). Two additional points for each monomer (B_P/L_ and C_P/L_ in Fig. 2) were chosen to provide for unambiguous definitions of the intermolecular angles and dihedrals.

Most monomers contain multiple functional groups, and separate interaction sites were created for each one. Interaction sites were classified into five categories as described below. Counts for each category are listed in Table 1.

1. General sites were created to broadly scan the full range of potential interactions a monomer can form. Exactly one general site was defined for all but three monomers (the exceptions are described below). The ligand set monomer points were defined simply and consistently: the primary site point A_L_ was taken from the geometric center of all heavy atoms, and B_L_ and C_L_ were defined arbitrarily from two other atoms, ensuring noncollinearity of the three points. For Na^+^ and Cl^−^, which are spherical, B_L_ and C_L_ were set as arbitrary points in space.
  More consideration was warranted for the protein set monomers. For some of the simpler protein monomers, A_P_, B_P_, and C_P_ were defined exactly as described above for the ligand set. However, for most of the others, the site points along with the allowed ranges of *θ*_*P*_ and *τ*_*P*_ (detailed in section “Allowed ranges for *θ* and *τ*” below) were defined to promote dimer configurations with contacts most likely to occur in the context of protein/ligand complexes.
2. Hydrogen bond donor (HBD) sites were defined for all hydrogen atoms bound to N, O, S, or sp-hybridized C atoms. For example, the HBD site for the methanol ligand monomer is shown in Fig. 2b.
3. Hydrogen bond acceptor (HBA) sites were defined for all O atoms and most N atoms, the exceptions being N atoms carrying a positive formal charge or trivalent N within or conjugated to a π-system (e. g., carboxamides, arylamines, or guanidines). For example, the HBA site for the carbonyl O of the N-methylacetamide protein monomer is shown in Fig. 2a.
4. Lewis base (LB) sites were defined for all fluorine atoms as well as hydrogen bond acceptors known to have strong, well-defined electron density (specifically, carbonyl O, heterocycle N, alkylamines, and N-oxides). Because of the large number of heterocycle N acceptors in the set of monomers, monocyclic but not bicyclic heterocycles were included when defining LB sites.
5. Lewis acid (LA) sites were defined for aryl chlorides and bromides and aromatic sulfur atoms, which contain a σ-hole opposite their bonds^32,33^. For heterocyclic sulfur, when the system was not symmetric, separate interaction sites were defined for each of the atoms bonded to the S. Also, sites were defined for carbonyl carbon atoms, which have been observed in favorable interactions with fluorine atoms or the oxygen atoms of other carbonyl groups in an orthogonal orientation^34^.

Three protein set monomers require special mention: the alanine and proline dipeptides and N-methylacetamide (compounds **8,** **9**, and **6** in Fig. 1, respectively). Peptide bonds are ubiquitous in proteins, so special care was taken to cover them thoroughly. The hydrogen bonding interactions of peptide bonds could be adequately represented with just the model of the peptide bond itself, N-methylacetamide. Consequently, HBD and HBA sites were created for N-methylacetamide but were not created for the two dipeptides because they were considered redundant. However, interactions with the π-cloud of the peptide face could be best represented by the dipeptide monomers, because they contain additional atoms off of the peptide plane to mimic amino acid side chains. Therefore, general sites were defined to favor partner placement adjacent to the peptide bond faces for the two peptide bonds in the alanine dipeptide as well as the proline N-containing peptide bond, but no general site was created for N-methylacetamide. Based on the same considerations, an LB site was created for the carbonyl O of only N-methylacetamide (not the dipeptides), and LA sites were created for only the dipeptide carbonyl carbons (not N-methylacetamide). Lastly, the two dipeptides also each had one site centered specifically on their side chain atoms, favoring partner placement to interact with the hydrophobic region distal to the backbone atoms.

For each interaction site, an appropriate range of acceptable values for *θ*_*P/L*_ and *τ*_*P/L*_ (Fig. 2a,b) were defined (detailed below). Structure files containing the optimized monomers with the locations of the interaction site points and ranges of *θ*_*P/L*_ and *τ*_*P/L*_ are available in the Splinter data repository^30^.

### Generation of random dimer configurations

To explore the potential energy surfaces of the interacting monomers, the interaction sites of the protein set were paired with those of the ligand set in sensible ways. First, all the protein general interaction sites were exhaustively paired with the ligand general interaction sites with the aim of broad coverage of the potential energy surface, especially capturing the nature of nonspecific van der Waals (vdW) contacts. In addition, to provide more sampling for hydrogen bond and Lewis acid/base interactions, which are generally both stronger and have more defined geometric preferences than vdW contacts, the protein HBD sites were exhaustively paired with the ligand HBA sites (and vice versa), and the protein LB sites were exhaustive paired with the ligand LA site (and vice versa). In total, there were 30416 of these “interaction site dimers” (so-called to distinguish them from “molecular dimers”; see below): 10098 general/general, 9693 protein HBD/ligand HBA, 4071 protein HBA/ligand HBD, 3174 protein LB/ligand LA, and 3380 LA protein/LB ligand dimers. Further details, including breakdowns by monomer charge state, are provided in Supplementary Tables S1–S5 and Table 2.

**Table 2:** Counts of all interaction site dimers classified by monomer charge.

Protein all/Ligand all	Ligand neutral	Ligand cation	Ligand anion	Ligand all
Protein neutral	14247	835	2401	**17483**
Protein cation	5292	147	1052	**6491**
Protein anion	5418	513	511	**6442**
**Protein all**	**24957**	**1495**	**3964**	**30416**

Each table element is the sum of equivalent elements in Supplementary Tables S1–S5.

For each interaction site dimer, multiple configurations were generated to adequately sample the interaction. To create an individual configuration, each monomer was held rigid at its optimized geometry, and the intermonomer orientation was set by randomly selecting values for the five angular internal coordinates (*θ*_*P*_, *θ*_*L*_, *τ*_*P*_, *τ*_*L*_, and *τ*_*PL*_; Fig. 2). Then, based on the orientation as well as a randomly generated value of the “vdW separation” *r* (defined below), *r*_*PL*_ was determined. In general, the probability distribution for the random sampling was not uniform, as described below.

Lastly, the position of each atom in the dimer was perturbed by a small displacement in a randomly selected direction and distance ≤0.1 Å. The purpose was to move each monomer slightly off its optimized geometry, which was found in previous work to be a necessary data augmentation strategy for ensuring that ML models derived from rigid monomers are robust with respect to minor changes in monomer geometry^11^.

This process was repeated 50 times for each interaction site dimer, yielding a total of 30416 × 50 = 1520800 total dimer configurations. Coordinates and calculated energy terms (see below) for all randomly generated configurations are available in the Splinter data repository^30^.

**Fig. 3:**
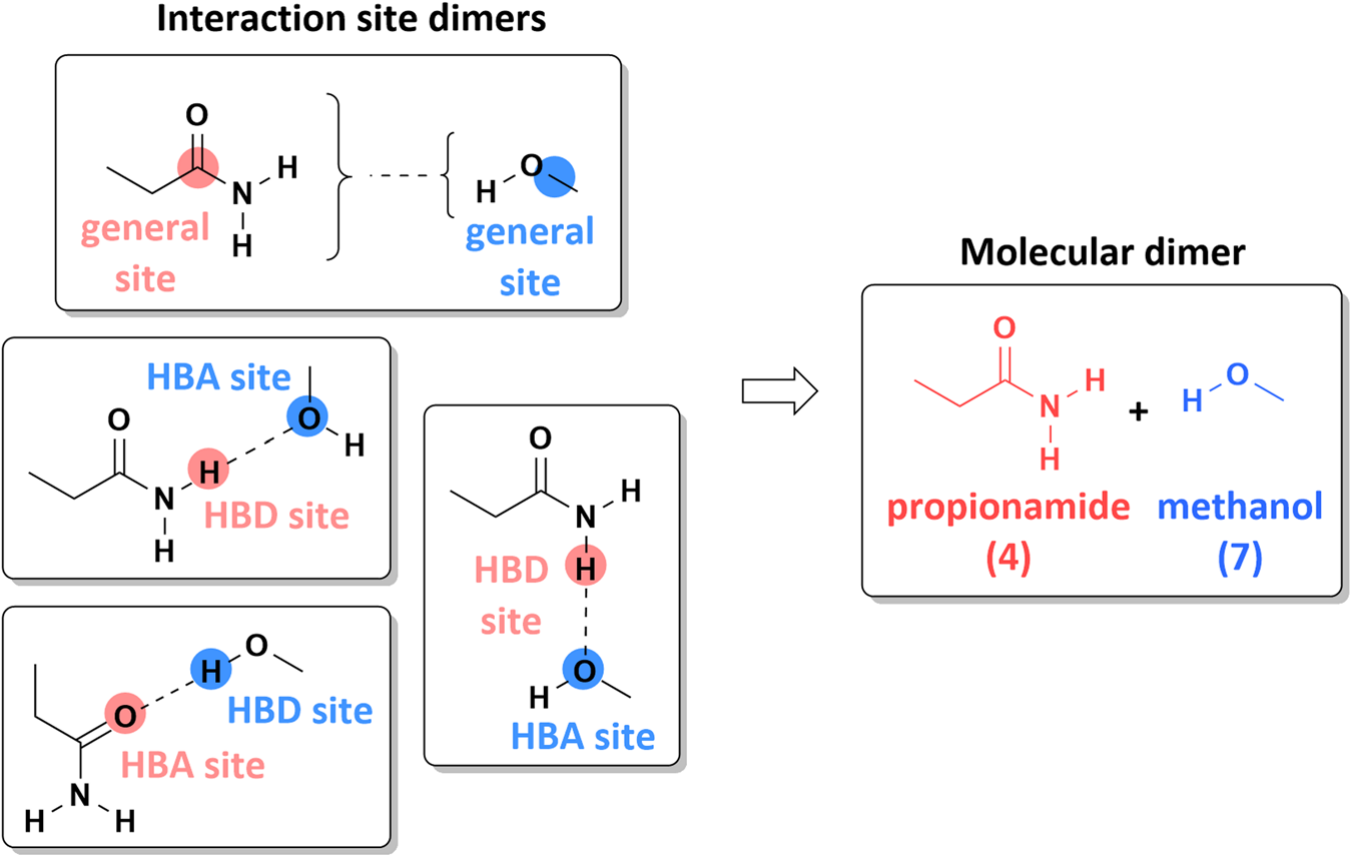
Four interaction site dimers assembled to form the propionamide/methanol molecular dimer. The interaction sites of the propionamide (red) and methanol (blue) monomers are highlighted.

Importantly, it should be noted that because most monomers have multiple interaction sites, a given protein monomer/ligand monomer pair could arise from various combinations of their interaction sites. For example, the propionamide protein monomer and methanol ligand monomer were paired together into four interaction site dimers (Fig. 3). However, each consists of the same monomers, so together these comprise the same “molecular dimer,” defined as a unique pair of monomers irrespective of their interaction sites. The 30416 interaction site dimers yielded a total of 9463 unique molecular dimers; counts are further detailed in Table 3. (Note that exhaustive combinatorial enumeration of 31 protein and 306 ligand monomers would produce 9486 molecular dimers; the slight reduction is due to the fact that several ligand monomers contained only a general interaction site and therefore were not paired with the protein monomer N-methylacetamide, which lacked a general site.) Molecular dimers included anywhere from 1 to 22 separate interaction site dimers, and correspondingly, 50 to 1100 random configurations. This heterogeneity reflects the need for more sampling of complex potential energy surfaces between monomers with multiple complementary functional groups.

**Table 3:** Counts of molecular dimers classified by monomer charge.

	Ligand neutral (250)	Ligand cation (23)	Ligand anion (33)	Ligand all (306)
Protein neutral (19)	4728*	436*	627	5791*
Protein cation (5)	1250	115	165	**1530**
Protein anion (7)	1750	161	231	**2142**
**Protein all (31)**	7728*	712*	**1023**	9463*

The count of monomers of each type (protein/ligand) and charge state is shown in parentheses. In general, the molecular dimer count is the product of the protein and ligand monomer count because in most cases, all pairwise combinations of monomers were used to generate molecular dimers. The exceptions (noted with *) are due to the fact that the N-methylacetamide monomer in the protein set (defined with HBD, HBA, LA, and LB interaction sites but no general interaction site) was not paired with any of 23 ligand monomers (22 neutral and one cationic) that contained only a general interaction site (e. g., hydrocarbons).

### Allowed ranges for *θ* and *τ *

In many cases, restricted ranges were set for an interaction site’s *θ* and *τ* values (Fig. 2), for two reasons. First, for some of the general interaction sites in protein monomers that mimic amino acid side chains, the ranges were restricted to promote the formation of interactions involving specific functional groups in the molecule and better recreate plausible protein-ligand binding interactions. The site points and the associated *θ* and *τ* ranges were set to favor the placement of binding partners adjacent to the monomer’s polar or aryl functional groups rather than its alkyl appendage. For example, the toluene fragment in the protein set (compound **10** in Fig. 1) represents a phenylalanine side chain. Because of the proximity of a phenylalanine’s Cβ atom to the protein backbone and its associated steric bulk, it is expected that ligands interact with its Cβ less frequently than its terminal phenyl side chain. Therefore, A_P_ was defined as the phenyl ring centroid, and the site points B_P_ and C_P_ and the ranges for *θ*_*P*_ and *τ*_*P*_ were defined to avoid configurations where the ligand is closest to the toluene’s methyl group rather than its phenyl ring. Similar considerations were applied to most other protein monomers.

Second, the motivation for creating the non-general (i. e., HBD, HBA, LB, or LA) interaction sites was to improve sampling of configurational space for these geometrically well-defined interactions. Therefore, the *θ* and *τ* ranges were restricted to explore only configurations that lie reasonably close to the low energy configurations.

For example, for all HBD sites, the range for *θ*, defining the D-H … A angle (e. g., Fig. 2b), was [90°, 180°]. The same range of [90°, 180°] was also used for *θ* in all carbonyl HBA sites (e. g., Fig. 2a), which defines the C = O … H angle in the hydrogen bond. Also, in carbonyls as LB sites, the range for *τ* was restricted to ≤ 45° with respect to the carbonyl plane (i. e., [−45°, 45°] and [135°, 225°]). In all cases, these ranges ensured that the configurational space of ensuing interaction site dimers was reasonably close to the optimal geometry. Note, however, that the allowed ranges were not set to be overly restrictive. For optimal coverage of the configurational space around these specific interactions that may have very deep minima, it was important to adequately sample even relatively poorly formed configurations.

The range for *τ*_*PL*_ was always completely unrestricted, i. e., (−180°, 180°].

### Allowed ranges for *r*

**Fig. 4:**
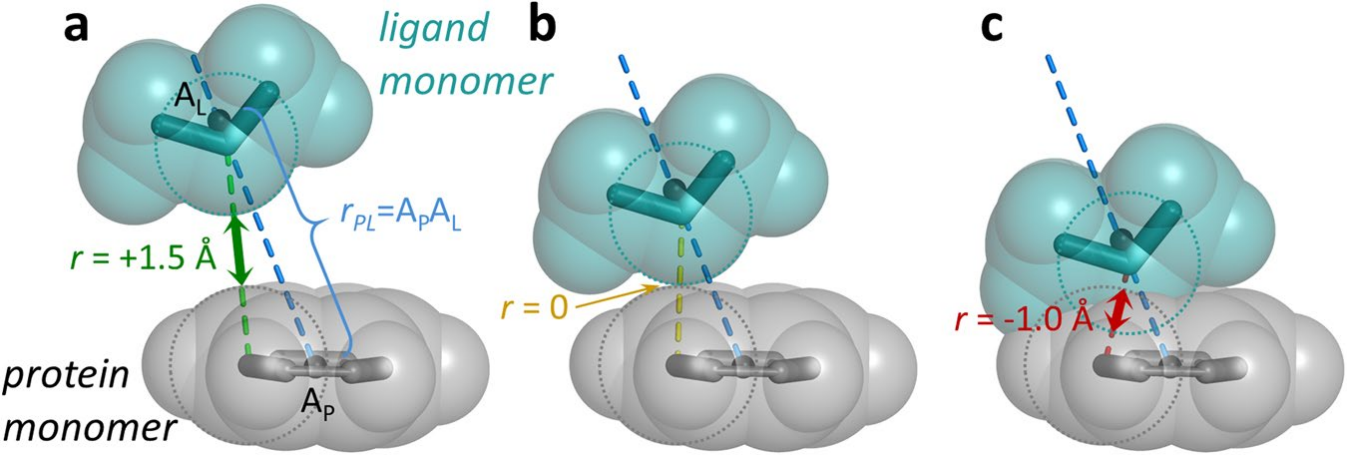
Description of van der Waals separation *r*. When finalizing a configuration, *r* is randomly selected, and the monomers are translated along the A_P_A_L_ vector (blue dashed line) such that the minimum distance between vdW surfaces for any pair of atoms (green, yellow, or red lines), one from each monomer, is *r*. (**a**) When *r* > 0, there is a separation between the surfaces. (**b**) When *r* = 0, the surfaces are tangent, in perfect vdW contact. (**c**) When *r* < 0, the surfaces overlap. Note the distinction between *r* and *r*_*PL*_. *r*_*PL*_ is the distance between the two primary interaction site points in the monomers (A_P_ and A_L_), which are not necessarily the same two atoms used in determining *r*. The specific value of *r*_*PL*_ is simply a geometric consequence of *r* and the angular orientation between the monomers.

Because of disparities in size and shape between monomers and the variety in the strength and nature of interactions that they could form, it was not practical to define a range for *r*_*PL*_, the absolute distance between monomer atoms as defined in Fig. 2, that would be suitable for all interaction site dimers regardless of orientation. Instead, the relevant distance metric that was used to complete the definition of each configuration is the “van der Waals (vdW) separation” *r*, defined by Eq. (1):

1 $$r=\mathop{\min }\limits_{p\in P,\,l\in L}\left({r}_{pl}-{r}_{vdW,p}-{r}_{vdW,l}\right)$$

where *r*_*pl*_ is the distance between the atomic centers and *r*_*vdW*_ the van der Waals radius of atoms *p* and *l* in protein and ligand monomers *P* and *L*, respectively. In other words, *r* is the closest approach between vdW surfaces of two monomers or, in the case of overlapping surfaces, the greatest extent of the vdW clash (Fig. 4). Using *r* as the distance metric allowed us to set consistent and meaningful ranges independently of the identities of the individual monomers.

The *r* ranges depended solely on the charges and the type of interaction sites (Table 4). For each type, the minimum allowed value for *r*, *r*_*min*_, was selected to ensure adequate sampling of complexes up to and even beyond the repulsive wall that arises from severe vdW clash. The maximum allowed value for *r*, *r*_*max*_, was selected to avoid dimer configurations that have such large intermonomer separation that their interaction energies are negligible. While it was desirable to obtain modest sampling of dimers with large *r* values (approaching *r*_*max*_), it was important to bias the sampling away from *r*_*max*_. This was due to the fact that widely separated monomers interact only weakly, so there is low configuration-dependent energy variance at large *r*. With a fixed total number of configurations, biasing away from *r*_*max*_ allows for more sampling at lower *r*, where the potential energy surface has higher variability. The desired bias away from *r*_*max*_ was accomplished by defining an intermediate distance *r*_*switch*_ between *r*_*min*_ and *r*_*max*_. The probability of sampling in the interval [*r*_*min*_, *r*_*switch*_] was made uniform, but the probability of sampling in the interval [*r*_*switch*_, *r*_*max*_] was made to decrease linearly to 0 (Supplementary Fig. S3).

**Table 4:** *r*_*min*_, *r*_*max*_, and *r*_*switch*_ values for different classes of interaction site dimers.

Charge states	Interaction site type	*r*_*min*_ (Å)	*r*_*max*_ (Å)	*r*_*switch*_ (Å)
charged/charged (opposite charges)		−1.8	5.0	1.5
charged/charged (like charges)		0.0	5.0	2.5
charged/neutral	general/general	−1.0	5.0	1.5
charged/neutral	HBD/HBA or LB/LA	−1.6	5.0	1.5
neutral/neutral	general/general	−1.0	3.0	1.0
neutral/neutral	HBD/HBA or LB/LA*	−1.3	3.0	1.0

*Includes cases where exactly one of the monomers is charged but the interaction site does not include the charged atom.

**Fig. 5:**
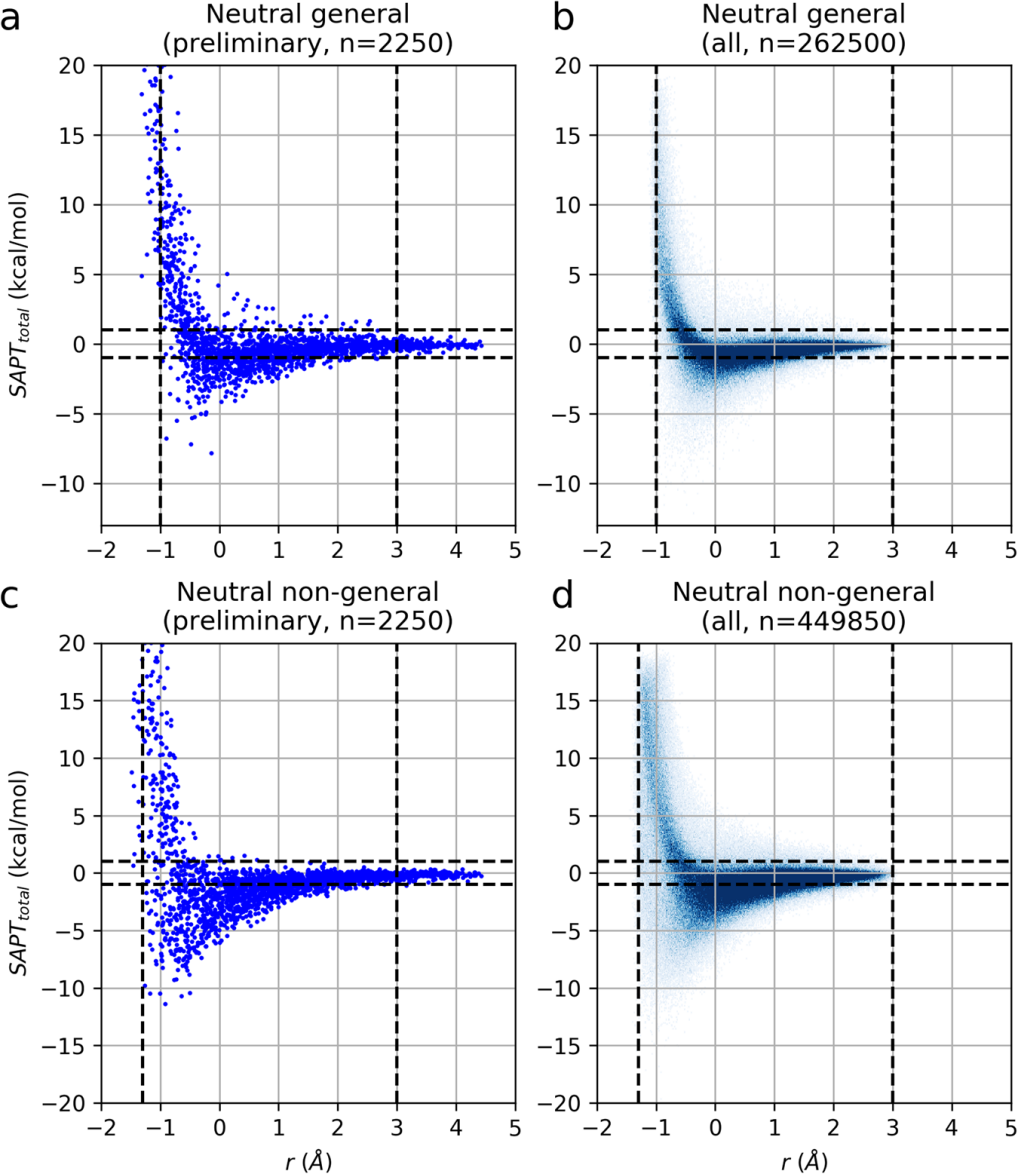
Dependence of SAPT0 energy on *r* for configurations of neutral interaction site dimers. (**a**) Preliminary set of 250 configurations for each of 9 pairs of general/general interaction site dimers. The left vertical dashed line at *r* = −1 Å indicates the value below which no configuration had a favorable (negative) energy. The right vertical dashed line at *r* =  +3.0 Å indicates the value above which no configuration had an energy exceeding ± 1 kcal/mol. Thus, these values were used to set *r*_*min*_ and *r*_*max*_, the lowest and highest values of the range allowed for *r* in generating random dimer configurations. (**b**) The same plot as panel a except the data is for all randomly generated configurations of general/general interaction site dimers in the final set, validating the suitability of the *r*_*min*_ and *r*_*max*_ values. (**c**) Plot similar to panel a except the data is for HBD/HBA interaction dimers; *r*_*min*_ and *r*_*max*_ were determined to be −1.3 and +3.0, respectively (vertical dashed lines). (**d**) The same plot as panel c except the data is for all randomly generated configurations of non-general (i. e., HBD/HBA and LA/LB) interaction site dimers, validating the suitability of the *r*_*min*_ and *r*_*max*_ values.

The values for *r*_*min*_, *r*_*max*_, and *r*_*switch*_ were determined empirically by an exploration with small subsets of representative interaction site dimers, for which several thousand configurations of each class with widely varying *r* were generated and their SAPT0 energies calculated as described below (Fig. 5a,c, Supplementary Figs. S4a,c, S5a,c, S6a,c). The data justifying these values are discussed in the “Technical Validation” section.

VdW radii values, required for the calculation of *r*_*PL*_ from the randomly-obtained *r* value, were taken from Alvarez^35^, except the value for Na^+^ was set to 1.50 Å, reduced from the published value of 2.50 Å (discussed further in the “Technical Validation” section).

### SAPT0 calculations

For each interaction site dimer configuration, SAPT0 calculations were performed with Psi4 (version 1.4)^36^ for both the jun-cc-pV(D + d)Z and aug-cc-pV(D + d)Z basis sets^37^. For each basis set, SAPT0 total energies and each of the four components energies (electrostatic, exchange, induction, and dispersion) were captured, as well as the total and component energies from the exchange-scaled variant (*s*SAPT0)^1^. Thus, there were a total of 20 calculated energy labels per configuration.

Configurations for which the total energy exceeded 20 kcal/mol (200 kcal/mol for repulsive charged dimers) were rejected because they were unlikely to be encountered in real-world applications. Because high energies invariably were the result of severe vdW clash, filtering by energy without some compensating consideration would bias the set of configurations away from those with low *r* values. The optimal way to avoid this bias would be to attempt new configurations with the same *r* value until one with an acceptable energy was obtained, but this approach was unfeasible because it would result in an infinite loop in situations where a given dimer has no possible configuration with acceptable energy at that *r* value. To avoid both of these issues, when a configuration was rejected due to energy, ensuing configurations were generated with a gradually expanding range of low *r* values until one was found to have an acceptable energy. Specifically, after the first failure, the range used to select *r* for the next configuration was [*r*_*min*_, *r*_*rejected*_ + 0.1], where *r*_*rejected*_ is the *r* value of the initial high-energy configuration. After each successive failure, the range for *r* was expanded by 0.1 Å, e. g., [*r*_*min*_, *r*_*rejected*_ + 0.2] for the second failure, [*r*_*min*_, *r*_*rejected*_ + 0.3] for the third, etc. This process was terminated upon generating one configuration with an acceptable energy, and the range for *r* was reset to [*r*_*min*_, *r*_*max*_] as usual to generate further configurations.

As shown in Supplementary Fig. S3, this process did not always completely prevent some degree of bias away from the lowest *r* values, i. e., there is relatively low actual representation of *r* values near *r*_*min*_ in Supplementary Fig. S3b,d, and e. However, this invariably coincided with an overrepresentation in the distribution for *r* values that were slightly larger but still quite low (with peaks centered at *r* = −1 Å or less), indicating that even when very low-*r*, high-energy complexes were eliminated, they were replaced with other complexes with *r* values nearly as low.

### Optimization of dimers

Random configurational sampling broadly captures the potential energy surface of interacting monomers, but it is likely to miss the fine details around the most crucial part of the surface—the local minima themselves. Therefore, optimizations of the dimers were performed to identify those local minima and supplement the random configurations.

Molecular dimers involving monomers of like charge were excluded from optimization, since the unfavorable electrostatic interactions would drive the monomers to an impractically large separation. For all others, optimizations were initiated from at least five starting configurations for each molecular dimer. The use of multiple starting points was intended to search for as many local minima as possible. For each molecular dimer, the starting configurations were obtained using the following multi-step sequence:

1. The set *S* of starting configurations for a given molecular dimer was initialized with one of the 50 randomly generated configurations from each of its associated interaction site dimers, specifically, the configuration with the lowest single-point interaction energy (SAPT0/jun-cc-pV(D + d)Z total energy), provided it was negative.
2. While *S* contains fewer than 5 configurations, iteratively add to *S* the remaining randomly generated configuration that has the highest root mean square deviation of coordinates of all heavy atoms (RMSD) from any configuration already present in *S*. In other words, if *R* is the set of all random configurations for the molecular dimer, and *a* denotes a configuration in *R* or *S* (with appropriate subscript), add to *S* the configuration *a*_*r*_ that maximizes the following function:

2 $$\mathop{\max }\limits_{{a}_{r}\in R-S}\,\mathop{\min }\limits_{{a}_{s}\in S}\,RMSD\left({a}_{r},\,{a}_{s}\right)$$

Step (1) identified low-energy starting configurations, potentially near different minima. Step (2) ensured that there were at least 5 configurations per molecular dimer, completing the set with configurations as diverse as possible. It should be noted that for additional diversity, step (2) was adapted slightly for molecular dimers consisting of only neutral monomers. In those cases, step (2) was performed for each interaction site dimer individually so that up to 5 configurations per *interaction site dimer* were added to *S*. For molecular dimers with at least one charged monomer, step (2) was performed only on the union of all interaction site dimers associated with the molecular dimer, so only up to 5 configurations per *molecular dimer* were added to S. Because most molecular dimers are associated with multiple interaction site dimers (e. g., Fig. 3), this difference in treatment tended to produce more starting configurations for neutral molecular dimers than for charged. Also, in some cases, step (2) added configurations with weak interaction energies and large intermonomer separation (*r*_*PL*_ > 3.6 Å), possibly occupying a flat part of the potential energy surface. The low gradient expected for such configurations could prevent the optimization from converging to a minimum, so even though these configurations were not immediately removed, additional configurations were added to *S* for the affected dimers to ensure that there were at least 5 starting configurations with *r*_*PL*_ < 3.2 Å. In total, 96679 optimizations were initiated for the 9117 molecular dimers, 72594 with neutral/neutral and 24085 with charged/neutral or charged/charged monomers.

Optimizations were performed with Psi4 (version 1.4)^36^, using the GeomeTRIC optimization engine^38^ at the B3LYP-D3/aug-cc-pV(D + d)Z level of theory^27,37,39^. Convergence criteria were set as shown in Table 5. Tighter criteria were used for configurations selected based on energy (in step 1 above), because presumably they were already relatively close to local minima compared to those selected based on structural diversity (step 2 above). Note that the atomic displacement criteria [both root mean square (RMS) and maximum] were set to such high values that they were essentially removed as convergence criteria because these thresholds exceeded the amount that occur in practice during an optimization. This was done to avoid spending time continuing to optimize weakly bound dimers, such as between two alkyl chains, that reach a broad but flat part of the potential energy surface. In such cases, the energies are very unlikely to change significantly once reaching the energy and gradient thresholds, even if the orientation between the two monomers has not yet reached the position of its true local minimum. Optimizations were run for at least 100 steps or until convergence.

**Table 5:** Convergence criteria for dimer optimization. The values are in atomic units.

Criterion	Energy-selected configurations	Diversity-selected configurations
ΔE	1e-6	1e-4
RMS gradient	3e-4	1.7e-3
Maximum gradient	4.5e-4	2.5e-3
RMS atomic displacement	1	1
Maximum atomic displacement	1	1

Calculations that terminated due to self-consistent field (SCF) convergence failure were restarted with different settings to obtain a different initial guess, first (a) from a generalized Wolfsberg-Helmholtz modification of the core Hamiltonian matrix^40^, then (b) by converging the SCF with the cc-pVDZ basis set^28^ and casting the orbital coefficients up to the final basis set, aug-cc-pV(D + d)Z^37^, and finally by both (a) and (b) simultaneously. Calculations that still failed were reattempted using more ideal initial geometries; instead of using the “perturbed” configurations (where all atoms were random moved immediately prior to SAPT0 energy calculation), each individual monomer was restored to its optimized geometry.

### Optimization post-processing

Structures resulting from the optimizations were eliminated from further consideration based on several criteria, including calculation failure due to SCF convergence, redundancy (having a heavy-atom RMSD <0.1 Å to another configuration of the same molecular dimer, taking into account reflections as well as monomer symmetry), excessive intermonomer separation (>3.6 Å, an indication that the configuration is probably not a true minimum), and in some cases, structural changes in the monomers. Structural changes were identified using a previously established method that required identical molecular graphs of heavy atoms from before and after optimization^41^. The graphs were determined from the initial monomer coordinates and final dimer by OpenBabel^42^, using the connectivity layer of the standard InChi. Stereochemistry, bond orders, and atomic charges were not considered in order to avoid inconsistencies arising from subtle changes in geometry when constructing the graph.

Optimizations that resulted in a change in the heavy-atom skeleton were eliminated. However, optimizations that resulted in a simple proton transfer from one monomer to another were not excluded. Instead, these optimized structures were recaptured by shifting the proton back to its original atom (by reducing the bond length to that in the optimized monomer) and using molecular mechanics (MacroModel version 12.8, part of Schrodinger software suite version 2020-2^43^, with the OPLS3e force field^44^) to minimize just the hydrogens while freezing the heavy atoms. These were retained as reasonable structures despite not being gas-phase minima.

Furthermore, all cases of proton transfer were manually inspected. If the newly formed molecular dimer was one of the standard dimers under study, it was added to the set of minima of the newly formed molecular dimer (after accounting for redundancy). If the proton transfer yielded a new molecular dimer that, despite being different from the original, was perfectly valid, it was retained in a separate set of “nonstandard” dimers. Further details are discussed in the “Technical Validation.”

In total, 78591 optimized configurations were obtained after filtering (77459 from “standard” and 1132 from “nonstandard” dimers). SAPT0 calculations were performed exactly as above using both their raw optimized coordinates as well as “perturbed” coordinates in which each atom was moved a random distance up to 0.1 Å in a random direction, completing successfully for 78515 configurations (77383 “standard” and all 1132 “nonstandard”). Coordinates and calculated energy terms for all 157030 (raw and perturbed) dimer configurations obtained by optimization are available in the Splinter data repository^30^.

### CCSD(T)/CBS energy calculations

Benchmark-quality reference interaction energies were computed with Psi4^36^ for ten of the configurations using focal-point estimates^45,46^ of coupled-cluster through perturbative triples [CCSD(T)]^47^ at the complete-basis-set (CBS) limit. Specifically, we performed two-point extrapolations^48^ of second-order perturbation theory (MP2) using the aug-cc-pVTZ and aug-cc-pVQZ basis sets^29^, and then added a correction for higher-order electron correlation computed as the difference between CCSD(T) and MP2 in the aug-cc-pVDZ basis; this procedure is denoted CCSD(T)/CBS(aug-cc-pV[TQ]Z; δ:aug-cc-pVDZ) for short. All energies included the Boys-Bernardi counterpoise correction^49^. The ten configurations were chosen as representatives of dimers with close contacts that have large differences in the induction/polarization component calculated by scaled and unscaled SAPT0/aug-cc-pV(D + d)Z (further discussed in the “Technical Validation”).

## Data Records

The Splinter dataset and supporting files are available in a Figshare data repository^30^. Each of the ~1.7M configurations is available in a file with standard .xyz format, with the first line consisting of the total number of atoms in the dimer, the second line containing a comma-separated alphanumeric string that contains all relevant non-coordinate information as detailed in Table 6, and all lines thereafter containing the element and Cartesian coordinates (in Å) for the atoms in the dimer. As shown in Table 6, four sets of total and component energies (in kcal/mol) for the dimer are included, both exchange-scaled and -unscaled values from two separate SAPT0 calculations [with basis sets jun- and aug-cc-pV(D + d)Z].

**Table 6:** Data contained in line 2 of Splinter .xyz files, with fields separated by commas.

Field index	Description	Basis set	Scaled/Unscaled
1	Dimer title and geometric parameters	N/A	N/A
2	Total charge	N/A	N/A
3	Monomer 1 charge	N/A	N/A
4	Monomer 2 charge	N/A	N/A
5	SAPT0 total energy	jun-cc-pV(D + d)Z	Unscaled
6	SAPT0 electrostatic energy	jun-cc-pV(D + d)Z	Unscaled
7	SAPT0 exchange energy	jun-cc-pV(D + d)Z	Unscaled
8	SAPT0 induction energy	jun-cc-pV(D + d)Z	Unscaled
9	SAPT0 dispersion energy	jun-cc-pV(D + d)Z	Unscaled
10	SAPT0 total energy	jun-cc-pV(D + d)Z	Scaled
11	SAPT0 electrostatic energy	jun-cc-pV(D + d)Z	Scaled
12	SAPT0 exchange energy	jun-cc-pV(D + d)Z	Scaled
13	SAPT0 induction energy	jun-cc-pV(D + d)Z	Scaled
14	SAPT0 dispersion energy	jun-cc-pV(D + d)Z	Scaled
15	SAPT0 total energy	aug-cc-pV(D + d)Z	Unscaled
16	SAPT0 electrostatic energy	aug-cc-pV(D + d)Z	Unscaled
17	SAPT0 exchange energy	aug-cc-pV(D + d)Z	Unscaled
18	SAPT0 induction energy	aug-cc-pV(D + d)Z	Unscaled
19	SAPT0 dispersion energy	aug-cc-pV(D + d)Z	Unscaled
20	SAPT0 total energy	aug-cc-pV(D + d)Z	Scaled
21	SAPT0 electrostatic energy	aug-cc-pV(D + d)Z	Scaled
22	SAPT0 exchange energy	aug-cc-pV(D + d)Z	Scaled
23	SAPT0 induction energy	aug-cc-pV(D + d)Z	Scaled
24	SAPT0 dispersion energy	aug-cc-pV(D + d)Z	Scaled
25	Number of atoms in monomer 1	N/A	N/A

The unit for reported energy values is kcal/mol.

The name of each file, which is the same as field 1 of the descriptor line, is derived from the identities of the interaction sites as well as the geometric parameters defining the dimer geometry. Specifically, the name was created by merging all of the following items, using an underscore as a separator: protein monomer name, protein interaction site type, ligand monomer name, ligand interaction site type, index, *r*, *θ*_*P*_, *τ*_*P*_, *θ*_*L*_, *τ*_*L*_, *τ*_*PL*_.

All .xyz files comprising Splinter are organized into the following directory tree. There is a single main directory for each molecular dimer that contains three subdirectories, named *random*, *opt_raw*, and *opt_perturb*. Files for all randomly generated configurations of the dimer, regardless of interaction site, are contained in directory *random*. Files of optimized configurations are in directory *opt_raw*, and files in which the optimized coordinates were perturbed slightly are in *opt_perturb*.

In addition to the .xyz files, each of these directories contains a supporting Python pickle (.pkl) file that contains a Python nested dictionary object with all energy information for the SAPT0 calculations. The energy values (in Hartrees) are stored in Pandas DataFrame objects^50^ accessible from the dictionary using the .xyz file name as the primary key and either “jun-cc-pV(D + d)Z” and “aug-cc-pV(D + d)Z” as the secondary key of the nested dictionary.

For molecular dimers for which no optimizations completed, there are no corresponding “opt_raw” and “opt_perturb” directories. For the “nonstandard” dimers (obtained only by H^+^ transfer during optimization), there is no corresponding “random” directory. The complete directory tree was processed by the Linux utilities tar and gzip to yield 11 large *.tar.gz files, 10 of which contain all the “standard” dimer configuration data and one with the “nonstandard” dimer configuration data.

In addition to the Splinter dataset files, the following supporting files are also available in the repository:

- SD files (one each for the protein and ligand monomer set) containing optimized structures of each monomer; properties associated with each structure include its SMILES string and classification as a member of the protein or ligand set. The name of each monomer was derived from its SMILES string (standardized by RDKit^51^) with all non-alphanumeric characters replaced with alphanumeric alternatives (e. g., every instance of ‘(‘ was replaced with ‘x’, ‘[‘ with ‘y’, etc.)
- SD files (one each for the protein and ligand monomer set) containing all the interaction sites; atoms A, B, and C of each site (see Fig. 2) as element I are appended to the optimized monomer; associated properties include the monomer’s SMILES string, its classification as protein or ligand, the type of interaction site, and the minimum and maximum values of the *θ* and *τ* ranges
- Script merge_monomers.py to create a series of Psi4 input files corresponding to randomly generated dimers from the interaction site SD files. The script relies on the Schrodinger Python API (https://www.schrodinger.com/pythonapi) for molecular structure processing and merging. It is compatible with all Schrodinger software versions dating back to at least 2020-1 up to and including at least 2023-2, which includes all releases in the three years prior to the time of publication. The Psi4 input files created by merge_monomers.py are compatible with Psi4 version 1.7, the latest stable release at the time of publication^36^.

## Technical Validation

### Comparison of the four energy methods in Splinter

Two SAPT0 variants (with and without exchange-scaling) were used with each of two basis sets [jun- and aug-cc-pV(D + d)Z] to generate the four methods of energy determination included in the Splinter data set. The errors for these methods with respect to benchmark-quality coupled-cluster complete-basis-set [CCSD(T)/CBS] calculations have been previously determined for the 4567 van der Waals dimers of small organic molecules comprising the HBC6, NBC10ext, S66x8, X31 × 10, SSI, and Ion43 test sets^52^. Mean absolute errors (MAEs) across the set depend on the basis set used and, to a lesser extent, the presence or absence of scaling: 0.57 for SAPT0/jun-cc-pVDZ, 0.59 for *s*SAPT0/jun-cc-pVDZ, 0.47 for SAPT0/aug-cc-pVDZ, and 0.42 kcal/mol for *s*SAPT0/aug-cc-pVDZ. In comparison, many of the more accurate density functional theory (DFT) methods for NCI energies exhibit MAEs in the range of 0.2–0.6 kcal/mol with the aug-cc-pVDZ basis set (without counterpoise correction) for the superset of the large sidechain-sidechain interaction (SSI) test set and the backbone-backbone interaction (BBI) test set^1,12^.

Of the four methods, *s*SAPT0/aug-cc-pVDZ appears to be the most accurate (MAE = 0.42 kcal/mol). However, we felt that the other three methods warranted inclusion in the Splinter data set based on considerations beyond accuracy. Even though SAPT0 or *s*SAPT0 with jun-cc-pVDZ is considerably less accurate than *s*SAPT0/aug-cc-pVDZ or SAPT0/aug-cc-pVDZ, especially for charged systems^52^, the use of the jun-cc-pVDZ basis set with SAPT0 calculations has been popular and remains relevant to the field based on an earlier study that found *s*SAPT0/jun-cc-pVDZ to yield a particularly favorable balance of speed and accuracy^1^. Also, with the aug-cc-pVDZ basis set, *s*SAPT0 is slightly more accurate than SAPT0, particularly for dimers with very close contacts such those found in doubly hydrogen bonded systems^1,52^. However, it should be noted that the exchange-scaling scheme is motivated primarily by practical experience, with the single parameter determined by fitting. Consequently, only SAPT0, not *s*SAPT0, may be considered a true *ab initio* approach, so SAPT0 is expected to be more suitable for general applications, even accounting for its slightly worse accuracy.

Based on these considerations, while there is sufficient value for all four methods to justify their inclusion in Splinter, we are primarily interested in the SAPT0/aug-cc-pV(D + d)Z energies. Comparison of the total and component energies across the entire data set between SAPT0/aug-cc-pV(D + d)Z and the other three methods, including distributions of their deviations, are shown in Supplemental Figs. S7–S11. In general, there is good agreement among all four methods. However, the exchange-scaling produces large differences, sometimes as large as 100 kcal/mol, in the induction/polarization component (and therefore the total energy) for a small fraction of dimers (<10000 out of the 1.6 million have energy differences >10 kcal/mol, most of them charged, all of them in very close contact with *r* <−0.5 Å; Supplemental Figs. S7, S10, and S12). To our knowledge, this is the first observation of such an effect in closely-bound dimers, so benchmark-level CCSD(T)/CBS interaction energies were obtained for ten representative dimers to quantify the accuracy of the four SAPT0 methods in such systems (Supplementary Table S6). Despite the tendency for the unscaled energies to be overbound compared to the benchmark values, they are more accurate because the scaled are underbound to an even larger degree. Altogether, the energy differences in closely-bound systems and better accuracy of the unscaled methods underscores our motivation to focus on the unscaled SAPT0/aug-cc-pV(D + d)Z energies.

### Validation of structures of optimized dimers

The 95580 optimizations that completed were carefully processed to ensure their validity. 14392 were eliminated for redundancy, 3663 for having an intermonomer separation of >3.6 Å, and 81 due to changes in the individual monomers’ Lewis structure. There were 1567 optimizations in which a proton was transferred between monomers in an acid-base reaction without any additional change in Lewis structure, invariably involving at least one and often two charged monomers. For these, the proton was returned to its original atom yielding a configuration of the original molecular dimer that was reasonable, although not a true gas-phase minimum. Fortuitously, in 19 proton-transfer cases, the optimization produced a molecular dimer that, while different from the original, was also in the “standard” set of 9463; 15 of these were nonredundant and were retained as separate minima for the new dimers. Altogether, the total number of optimized structures in the standard set of molecular dimers was 77459. Of the 9463 standard molecular dimers, at least one minimum was obtained for 9104; the others were missing either because they contained monomers of like charge (and therefore were not even attempted) or had no calculations pass all filters of the process.

The 1548 optimizations that resulted in proton transfer but did not form a standard molecular dimer were closely inspected. Some involved species that were clearly nonphysical in the context of biochemical systems, such as high-energy tautomers or unreasonable acids or bases (e. g., amide N^−^ conjugate base). However, 1132 appeared reasonable and were retained as potentially useful optima despite consisting of “nonstandard” dimers. A total of 400 different nonstandard molecular dimers were obtained, each contributing 1–12 configurations to Splinter.

### Validation of *r*_*min*_ and *r*_*max*_ for different interaction types

The values for *r*_*min*_ and *r*_*max*_ were determined empirically by an exploration with small subsets of representative interaction site dimers, for which several thousand configurations of each class with widely varying *r* were generated and SAPT0 energies calculated. For dimers consisting of neutral monomers, Fig. 5 shows the SAPT0 energies plotted against *r* for the exploratory studies (left panels) as well as the complete set of the randomly generated configurations (right panels), both for general interaction site dimers (panels a and b) and HBD/HBA and LB/LA interaction site dimers (panels c and d). Supplementary Figs. S4–S6 show similar plots for dimers involving one or more charged monomers.

Due to the repulsive nature of vdW clash, most complexes with low *r* had very large and unfavorable energies. For each interaction site dimer type, the minimum allowed *r* value, *r*_*min*_, was set to a value just low enough to include all favorable complexes in the exploratory set. In other words, no exploratory configuration with an *r* value below *r*_*min*_ had a negative SAPT0 energy. The data for all randomly generated configurations demonstrate that *r*_*min*_ was set appropriately. Note that for the repulsive charged/charged dimers, no configuration ever had favorable energy, so *r*_*min*_ was set to 0 (Supplementary Fig. S5a).

**Fig. 6:**
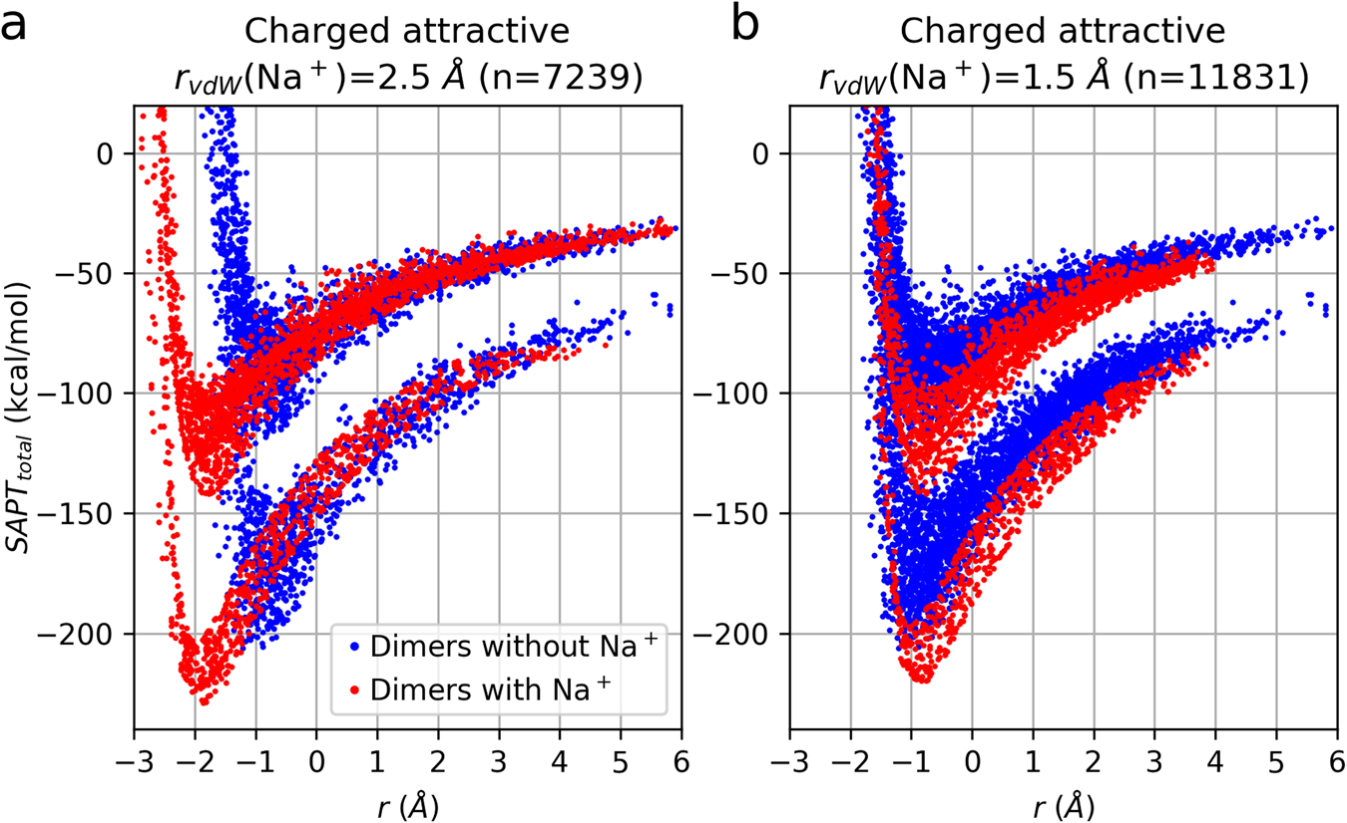
Dependence of SAPT0 energy on *r* for configurations of attractive charged dimers assuming different values for the vdW radius of Na^+^. (**a**) Energy vs. *r* plots for dimer configurations with and without Na^+^ (red and blue points, respectively) that were generated assuming the published vdW radius of 2.5 Å for Na^+^. All curves have similar shapes, but those with Na^+^ appear to be left-shifted by ~1 Å. (**b**) Similar data as in panel a, except configurations were generated after modifying the vdW radius of Na^+^ to 1.5 Å. The non-Na^+^ and Na^+^ curves are much more closely overlapping.

Na^+^ complexes require special mention. Using the published value of 2.5 Å for the vdW radius of Na^35^, the energy vs. *r* plot for the resultant configurations have the same shape as for other complexes, except it appears to be left-shifted by ~1 Å (Fig. 6a). When the Na^+^ vdW radius was decreased to 1.5 Å, the data for the exploratory set no longer contained this discrepancy (Fig. 6b), so this modified value was used for the full set. This modification is justified because the vdW radii are useful in this work only to the extent that they provide consistent sampling of the potential energy surfaces of all dimers, and only the modified value for Na^+^ allows for that consistency. Note that the values of the vdW radii underlying the generation of configurations have no effect whatsoever on the ensuing SAPT0 energies.

The maximum allowed *r* value, *r*_*max*_, was set to limit the generation of negligibly interacting, widely separated dimer configurations. The value for *r*_*max*_ was selected as the approximate upper bound for all exploratory configurations with SAPT0 energies exceeding ±1 kcal/mol. In other words, above this threshold, all configurations had very weak interaction energies, between −1 and +1 kcal/mol (Fig. 5a,c)

Because of the long-range nature of Coulombic forces, charged/charged dimers (whether attractive or repulsive) required special consideration. The interaction energy for two elementary charges would not fall below the 1 kcal/mol threshold until the intermonomer separation exceeded 300 Å, far beyond any reasonable range of *r*. Therefore, for charged dimers, when determining the interaction energy with respect to the 1 kcal/mol threshold, the electrostatic component of the SAPT0 total energy was excluded, i. e., only the exchange, induction, and dispersion components were summed. This led to an *r*_*max*_ value of 5.0 Å, similar to the 3.5-Å *r*_*max*_ value for neutral dimers and much more palatable for dimer construction (Supplementary Figs. S4c, S5c).

The plots of energy vs. r for the entire randomly generated dataset are very similar to those from the exploratory sets. At the low end of the range of *r*, there are very few configurations with favorable energies, and at the high end, almost all configurations have energies between −1 and 1 kcal/mol (Fig. 5b,d and Supplementary Figs. S4b, S4d, S5b, S5d, S6b, and S6d). This demonstrates the suitability of the *r*_*min*_ and *r*_*max*_ values.

### Supplementary information

Supplementary Information

## Data Availability

Script merge_monomers.py, used to create the Psi4 input files that were used for SAPT0 energy calculations, is available as part of the figshare repository containing the Splinter dataset^30^.
