# Supplementary material for: A quantum chemical interaction energy dataset for accurately modeling protein-ligand interactions
Source: Sci Data. 2023 Sep 12;10:619. doi: 10.1038/s41597-023-02443-1 (PMC10497680; doi:10.1038/s41597-023-02443-1)
Supplement: Supplementary file 1 — Supplementary Information [file 41597_2023_2443_MOESM1_ESM.pdf]

Supplementary information for
A quantum chemical interaction energy dataset for accurately modeling protein-ligand interactions

Steven A. Spronk[1], Zachary L. Glick[2], Derek P. Metcalf[2], C. David Sherrill[2], and Daniel L. Cheney[1]
1. Molecular Structure and Design, Bristol Myers Squibb Company, P. O. Box 5400, Princeton, NJ 08543, USA
2. Center for Computational Molecular Science and Technology, School of Chemistry and Biochemistry, and School of Computational Science and Engineering, Georgia Institute of Technology, Atlanta, GA 30332-0400, USA

Table of contents

Supplementary Fig. S1: Protein set molecular fragments 2
Supplementary Fig. S2: Ligand set molecular fragments 3
Supplementary Table S1: Counts of general/general interaction site dimers classified by monomer charge 4
Supplementary Table S2: Counts of protein HBD/ligand HBA interaction site dimers classified by monomer charge 4
Supplementary Table S3: Counts of protein HBA/ligand HBD interaction site dimers classified by monomer charge 4
Supplementary Table S4: Counts of protein LB/ligand LA interaction site dimers classified by monomer charge 4
Supplementary Table S5: Counts of protein LA/ligand LB interaction site dimers classified by monomer charge 4
Supplementary Fig. S3: Probability distribution for selection of r for different classes of interaction site dimers 5
Supplementary Fig. S4: Dependence of SAPT0 energy on r for configurations of attractive charged dimers 6
Supplementary Fig. S5: Dependence of SAPT0 energy on r for configurations of repulsive charged dimers 7
Supplementary Fig. S6: Dependence of SAPT0 energy on r for configurations of neutral/charged dimers 8
Supplementary Fig. S7: Total SAPT0 energies calculated with different basis sets and scaling treatment compared to SAPT0/aug-cc-pV(D+d)Z 9
Supplementary Fig. S8: Electrostatic component of SAPT0 energies calculated with different basis sets and exchange-scaling treatment compared to SAPT0/aug-cc-pV(D+d)Z 10
Supplementary Fig. S9: Exchange-repulsion component of SAPT0 energies calculated with different basis sets and exchange-scaling treatment compared to SAPT0/aug-cc-pV(D+d)Z 11
Supplementary Fig. S10: Induction/polarization component of SAPT0 energies calculated with different basis sets and exchange-scaling treatment compared to SAPT0/aug-cc-pV(D+d)Z 12
Supplementary Fig. S11: London dispersion component of SAPT0 energies calculated with different basis sets and exchange-scaling treatment compared to SAPT0/aug-cc-pV(D+d)Z 13
Supplementary Fig. S12: Dependence on r of the difference in the induction/polarization component of SAPT0 energies with and without exchange-scaling treament 14
Supplementary Table S6: Interaction energies for representative dimers with close contacts 15

Supplementary Fig. S1: Protein set molecular fragments. Groupings are by charge (black box: neutral, red box: anionic, blue box: cationic)

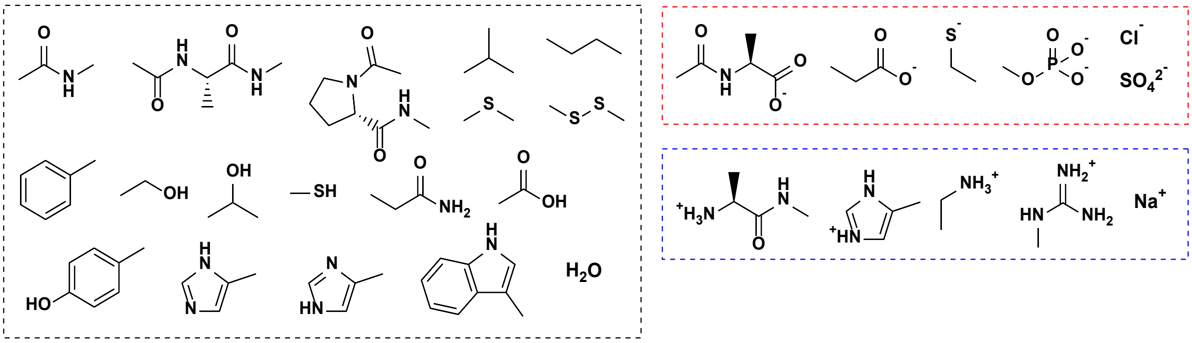

Supplementary Fig. S2: Ligand set molecular fragments. Groupings are by charge (black boxes: neutral, red box: anionic, blue box: cationic), with neutral fragments classified more narrowly based on chemical structure.

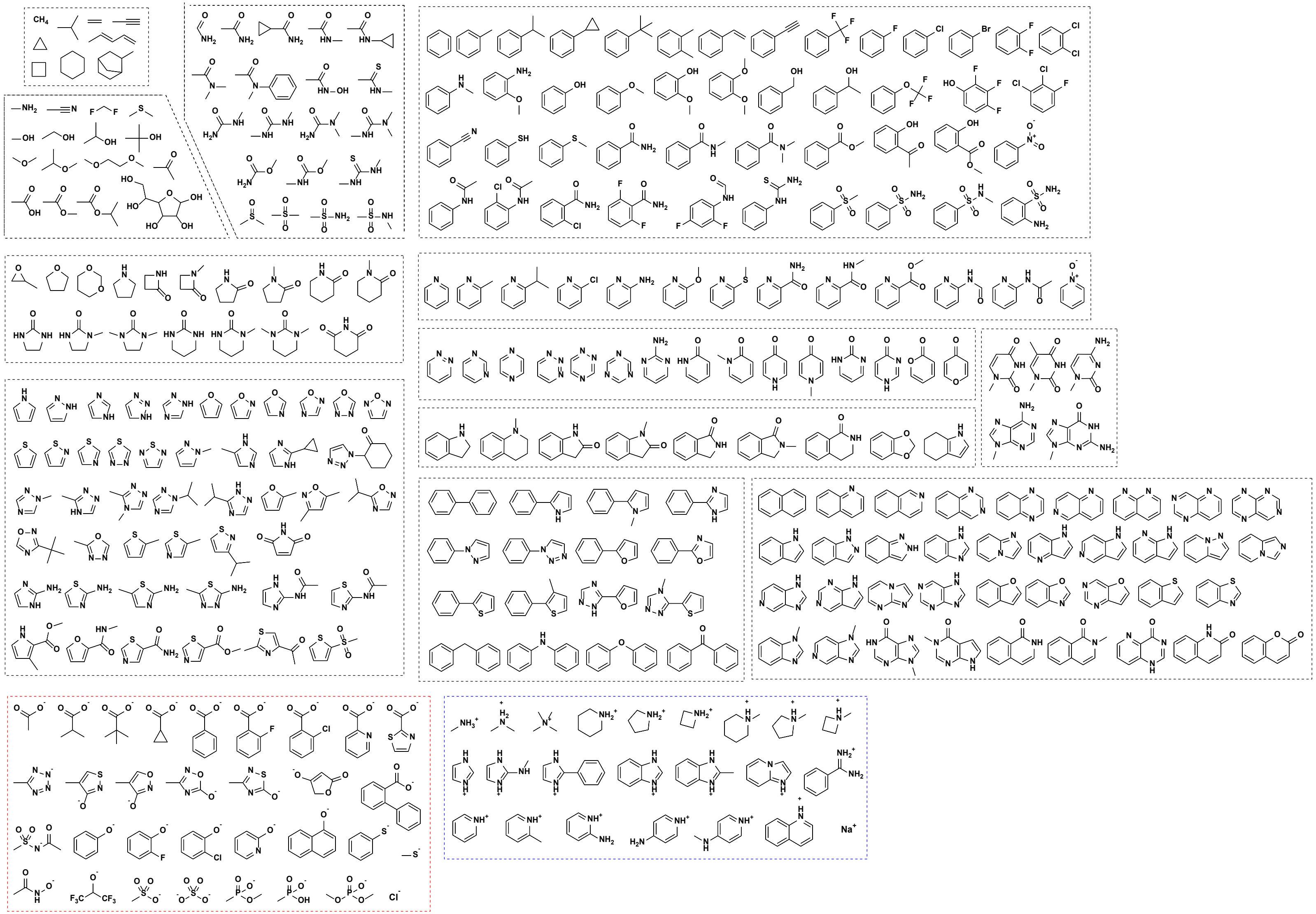

Supplementary Table S1: Counts of general/general interaction site dimers classified by monomer charge. The count of interaction sites of each type (protein/ligand) and monomer charge state is shown in parentheses, here as well as in Supplementary Tables S2-S5.

**Protein general/Ligand general**	Ligand neutral (250)	Ligand cation (23)	Ligand anion (33)	**Ligand all (306)**
Protein neutral (21)	5250	483	693	**6426**
Protein cation (5)	1250	115	165	**1530**
Protein anion (7)	1750	161	231	**2142**
**Protein all (33)**	**8250**	**759**	**1089**	**10098**

Supplementary Table S2: Counts of protein HBD/ligand HBA interaction site dimers classified by monomer charge.

**Protein HBD/Ligand HBA**	Ligand neutral (293)	Ligand cation (0)	Ligand anion (66)	**Ligand all (359)**
Protein neutral (14)	4102	0	924	**5026**
Protein cation (12)	3516	0	792	**4308**
Protein anion (1)	293	0	66	**359**
**Protein all (27)**	**7911**	**0**	**1782**	**9693**

Supplementary Table S3: Counts of protein HBA/ligand HBD interaction site dimers classified by monomer charge.

**Protein HBA/Ligand HBD**	Ligand neutral (143)	Ligand cation (32)	Ligand anion (2)	**Ligand all (177)**
Protein neutral (11)	1573	352	22	**1947**
Protein cation (1)	143	32	2	**177**
Protein anion (11)	1573	352	22	**1947**
**Protein all (23)**	**3289**	**736**	**46**	**4071**

Supplementary Table S4: Counts of protein LB/ligand LA interaction site dimers classified by monomer charge.

**Protein LB/Ligand LA**	Ligand neutral (129)	Ligand cation (0)	Ligand anion (9)	**Ligand all (138)**
Protein neutral (10)	1290	0	90	**1380**
Protein cation (1)	129	0	9	**138**
Protein anion (12)	1548	0	108	**1656**
**Protein all (23)**	**2967**	**0**	**207**	**3174**

Supplementary Table S5: Counts of protein LA/ligand LB interaction site dimers classified by monomer charge.

**Protein LA/Ligand LB**	Ligand neutral (254)	Ligand cation (0)	Ligand anion (84)	**Ligand all (338)**
Protein neutral (8)	2032	0	672	**2704**
Protein cation (1)	254	0	84	**338**
Protein anion (1)	254	0	84	**338**
**Protein all (10)**	**2540**	**0**	**840**	**3380**

Supplementary Fig. S3: Probability distribution for selection of r for different classes of interaction site dimers. Each panel shows the probability distribution (thick black line) and the actual distribution of r values among the randomly generated configurations in Splinter (thin blue bars) for different classes as indicated by the panel's title. All r_{min}, r_{max}, and r_{switch} values are labeled.

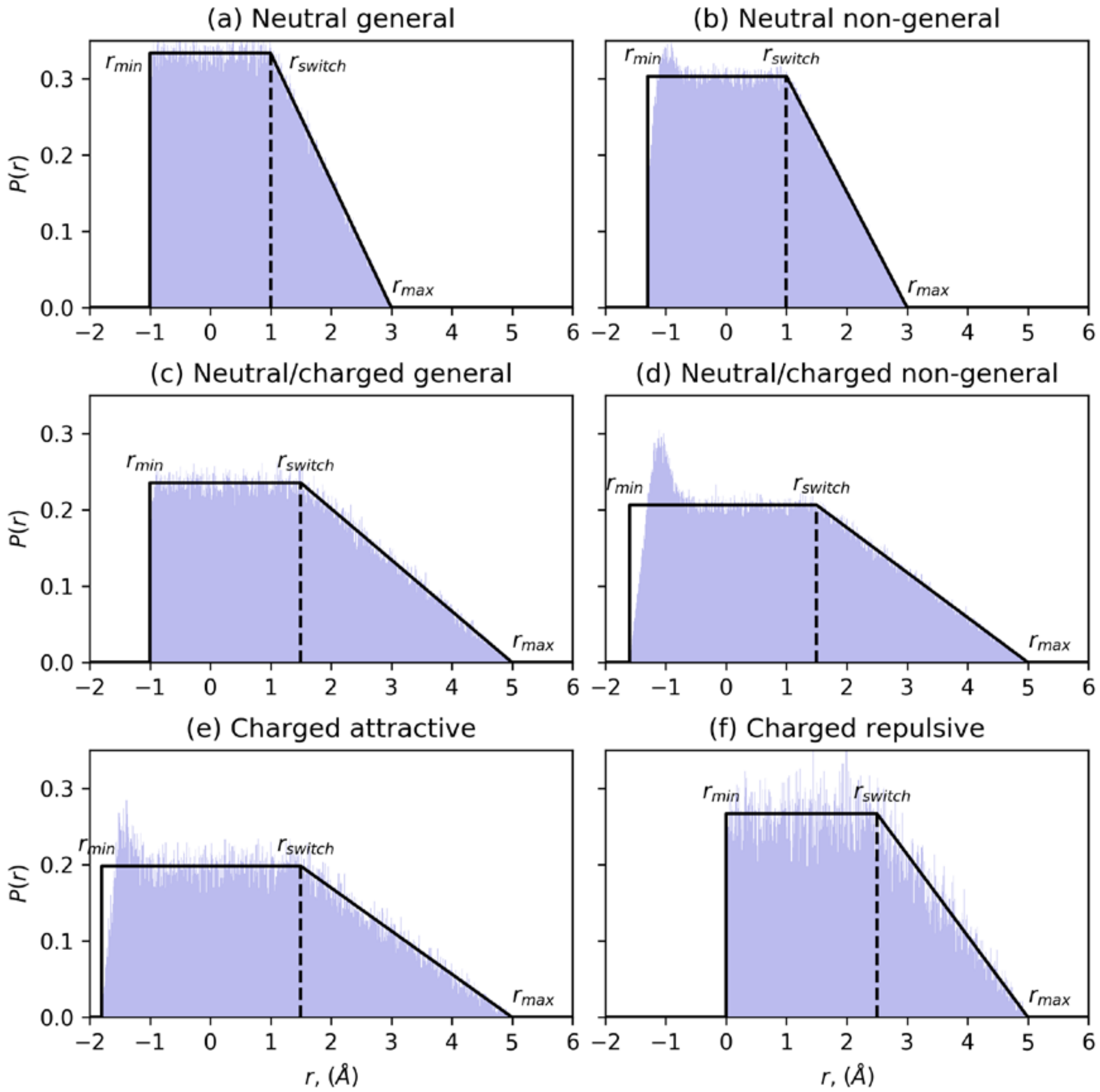

Supplementary Fig. S4: Dependence of SAPT0 energy on r for configurations of attractive charged dimers. (a) Preliminary set of 11831 configurations of general/general interaction site dimers. The vertical lines indicate the values of r_{min} and r_{max} eventually selected. r_{min} was set to -1.8 Å because in the preliminary set, this value is the lower bound for all configurations with favorable (negative) energy. Due to the long range nature of electrostatic attraction, the energy remains very favorable out to the 6 Å limit of the data in this experiment. (b) The same plot as in panel a except the data is for all randomly generated configurations of general interaction site dimers, validating the suitability of r_{min}. The dashed blue curve is of functional form $1/(r+r_{vdW})$ to represent the classical electrostatics energy curve; the reasonable fit of all data to this curve for r > 4.0 Å indicates that electrostatics component of the SAPT0 energy begins to overwhelm the other three components at this r value. (The value for r_{vdW} for the dashed curve is empirically determined to be 2.625 Å, and as such is the apparent mean vdW radius of all charged monomers in this study.) (c) Plot similar to panel a except the energy value plotted on the y-axis is the electrostatics component subtracted from the SAPT0 total energy. Using these data, r_{max} was determined to be 5 Å, because this is the upper bound for configurations with a total contribution from the non-electrostatics components of at least ±1 kcal/mol. This is another visualization of the fact that electrostatics component dominates the others at r > 4 Å. (d) The same plot as panel c except the data is for all randomly generated configurations of general interaction site dimers, validating the suitability of r_{max}.

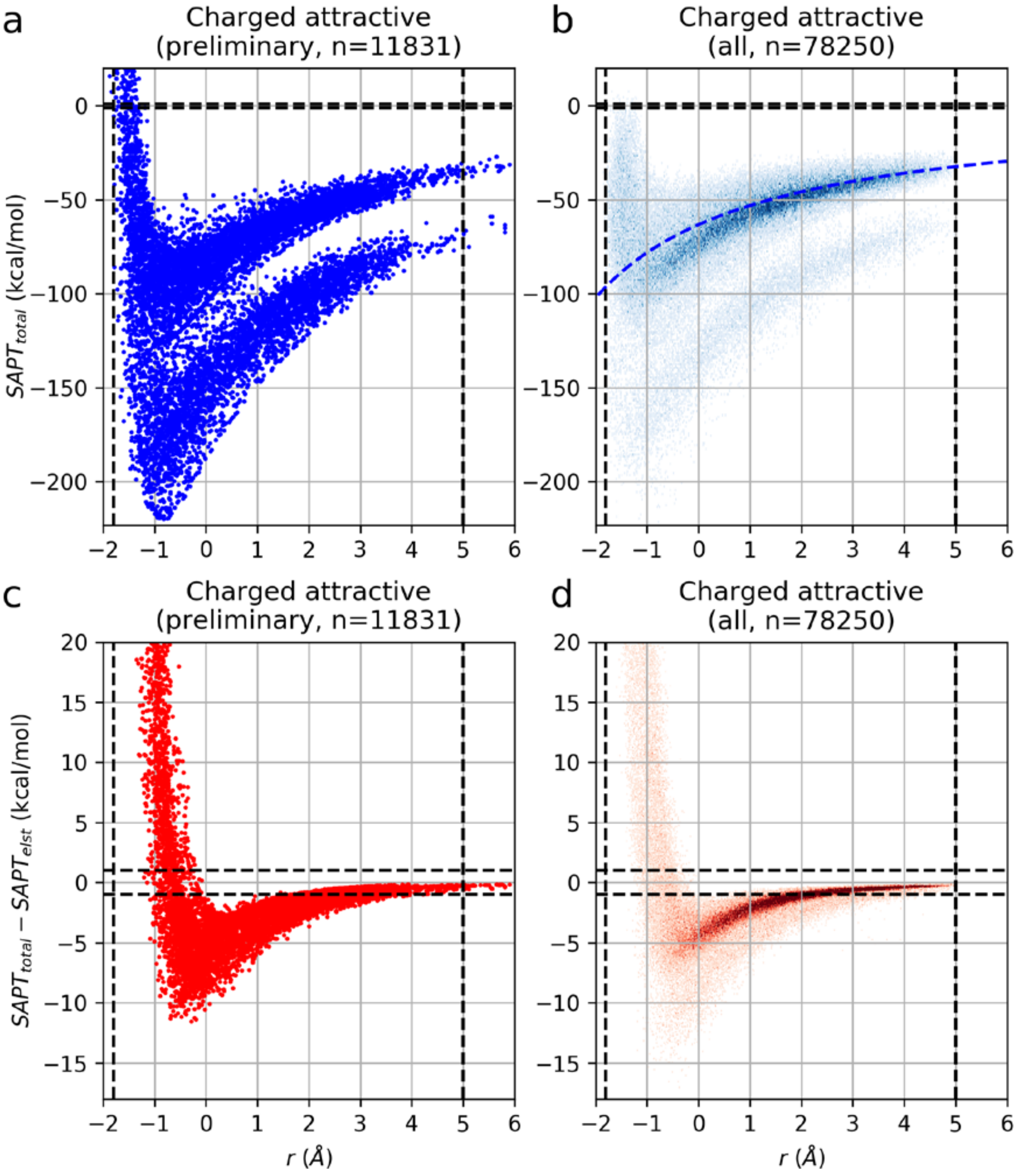

Supplementary Fig. S5: Dependence of SAPT0 energy on r for configurations of repulsive charged dimers. All panels are analogous to those in Supplementary Fig. S4, except the dimers here contain charged monomers with like charges instead of opposite charges. r_{max} was determined to be +5.0 Å. Note that all configurations had very unfavorable energies at all r. This precludes the use of the typical definition used to determine r_{min}, which is the lower bound of configurations with negative energy, so r_{min} was arbitrarily set to 0.

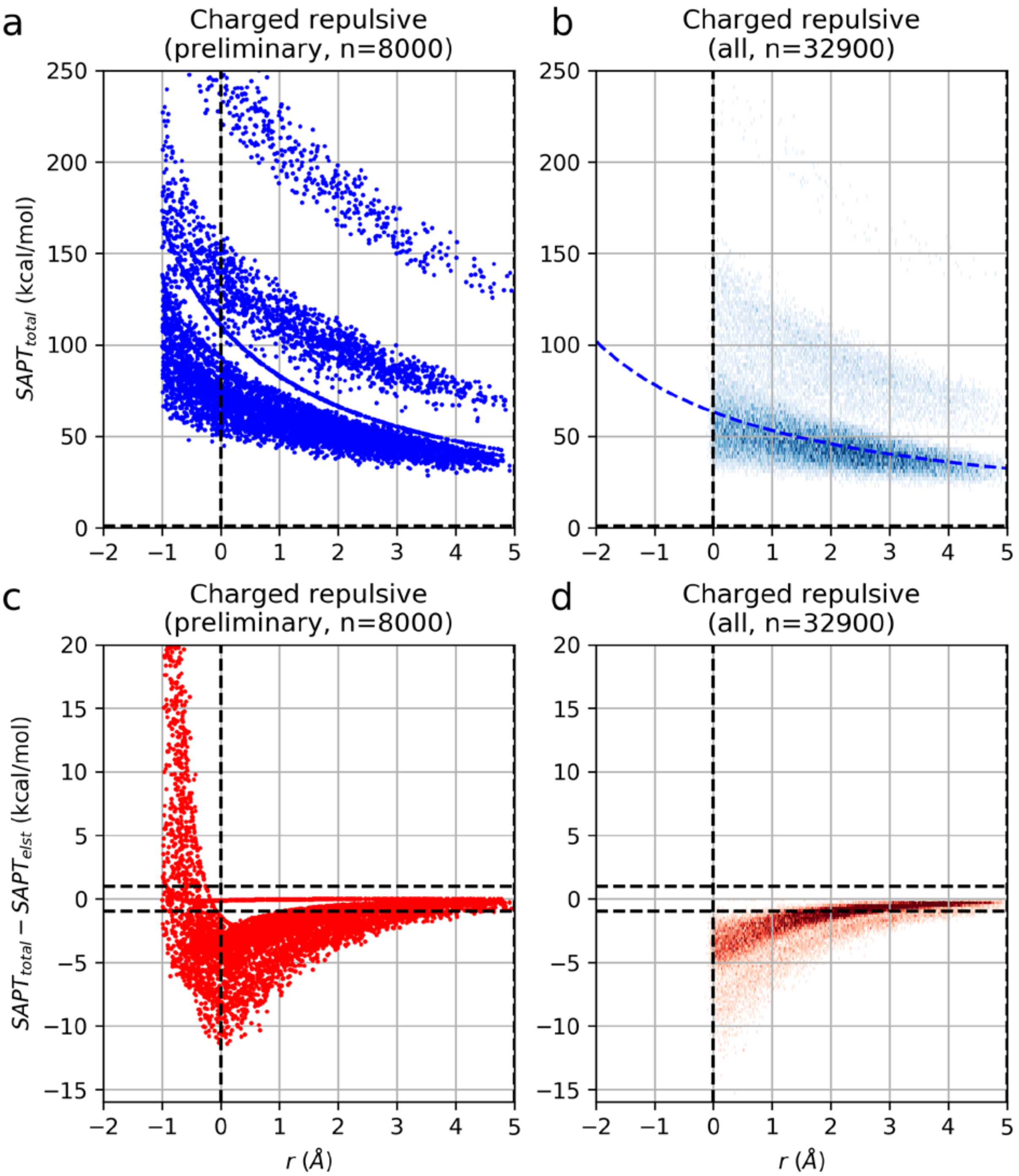

Supplementary Fig. S6: Dependence of SAPT0 energy on r for configurations of neutral/charged dimers. All panels are analogous to those in Supplementary Fig. S4, except the dimers here contain one neutral and one charged monomer (of either sign). r_{min} and r_{max} were determined to be -1.6 and +5.0, respectively.

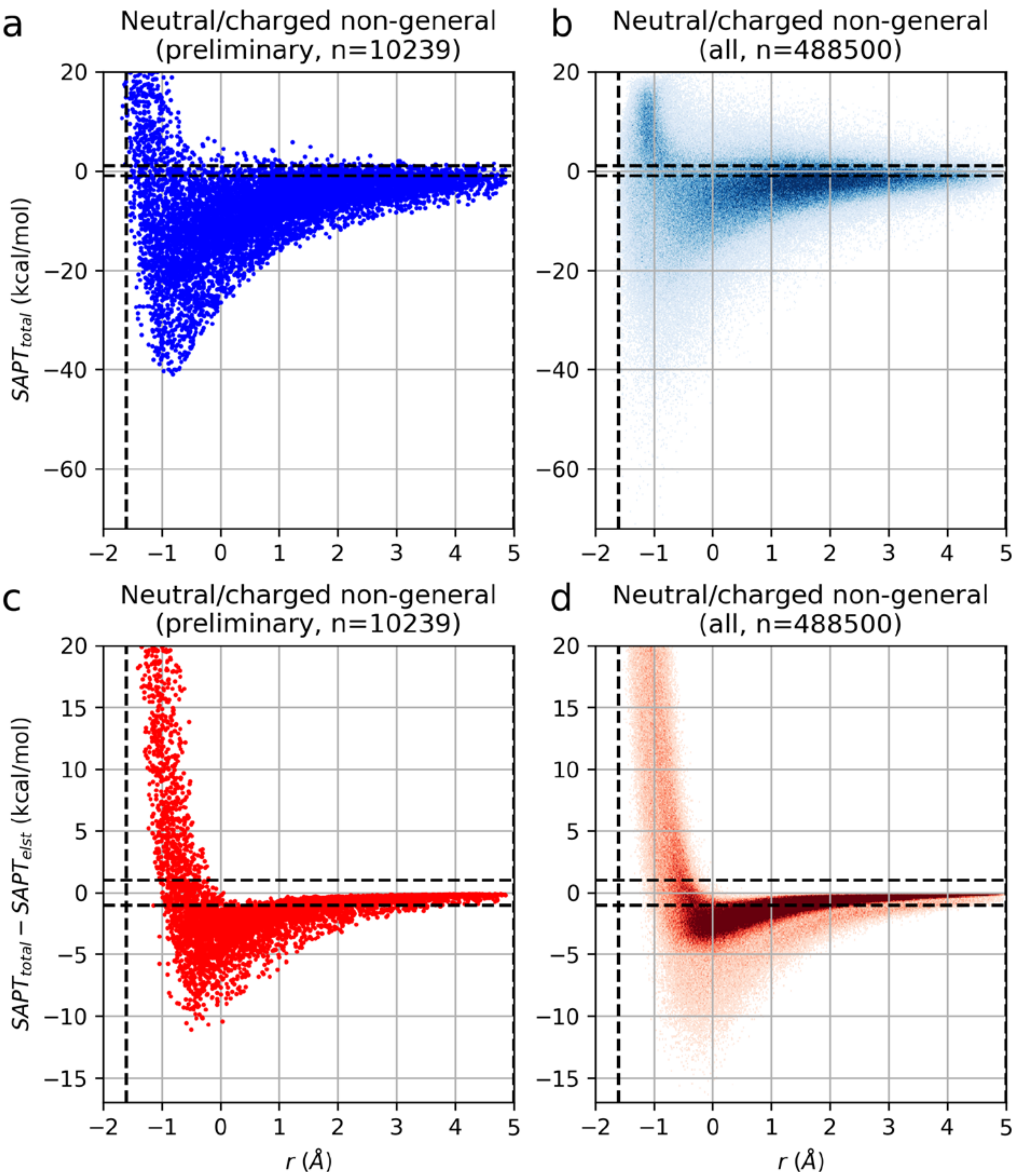

Supplementary Fig. S7: Total SAPT0 energies calculated with different basis sets and exchange-scaling treatment compared to SAPT0/aug-cc-pV(D+d)Z. Total interaction energies calculated by SAPT0/jun-cc-pV(D+d)Z (a), sSAPT0/jun-cc-pV(D+d)Z (c), and sSAPT0/aug-cc-pV(D+d)Z (e) are plotted against SAPT0/aug-cc-pV(D+d)Z as a 2D histogram (with bin widths of 1 kcal/mol along both dimensions); more intense colors reflect higher populations. Panels b, d, and f show histograms of the signed deviations of the energy values, with bin widths of 0.1 (b) or 1 kcal/mol (d, f). Blue dots/lines represent dimers of two neutral monomers; red represent dimers with at least one charged monomer.

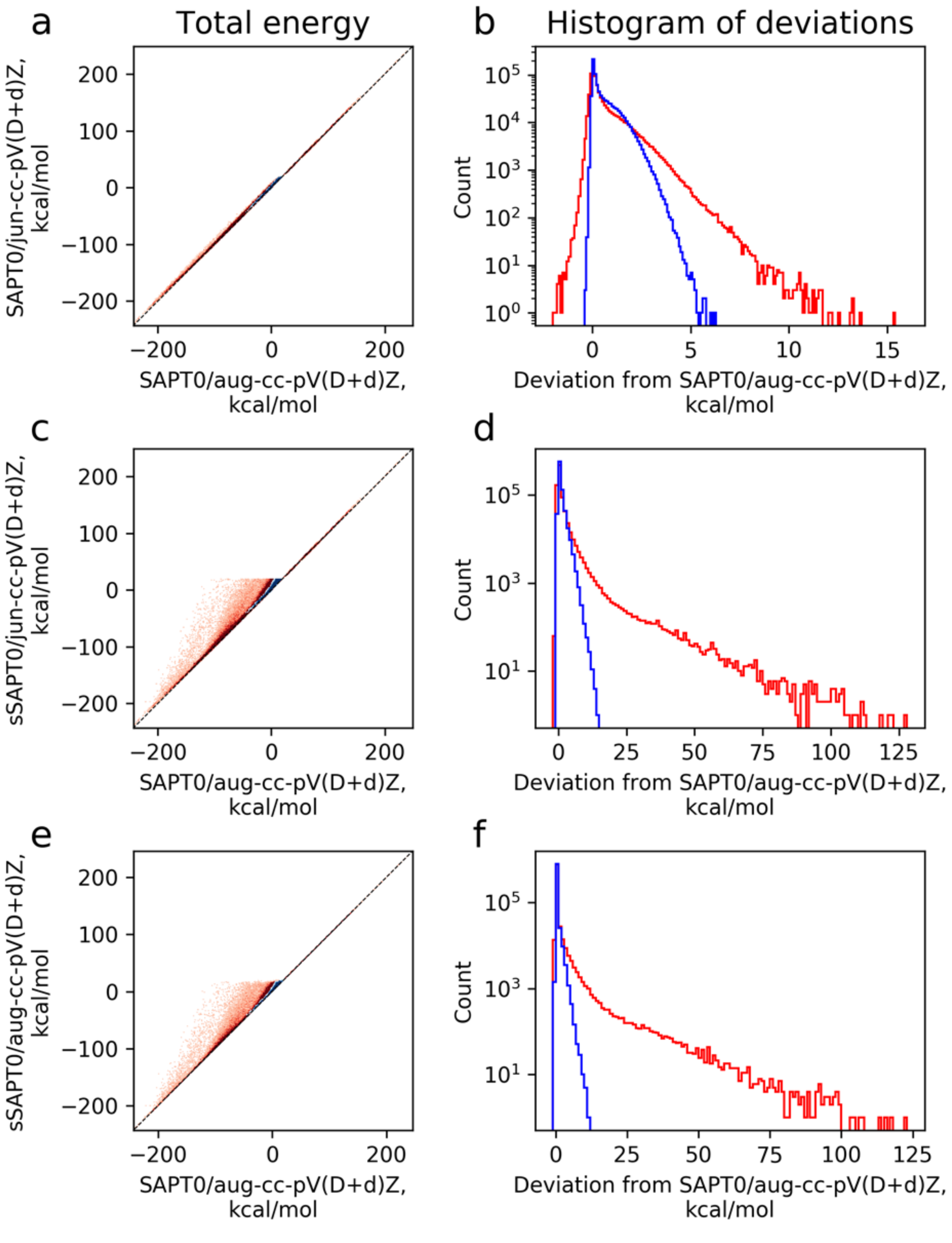

Supplementary Fig. S8: Electrostatic component of SAPT0 energies calculated with different basis sets and exchange-scaling treatment compared to SAPT0/aug-cc-pV(D+d)Z. All panels are analogous to those in Supplementary Fig. S7, except the energies here are just the electrostatic component of the SAPT0 interaction energies. Bin widths are 2 kcal/mol in both dimensions for 2D histograms (a, c, e) and 0.1 kcal/mol for 1D histograms (b, d, f). Note that the exchange-scaling algorithm does not modify this component, so the scaled and unscaled SAPT0/aug-cc-pV(D+d)Z energies are in perfect agreement (e, f).

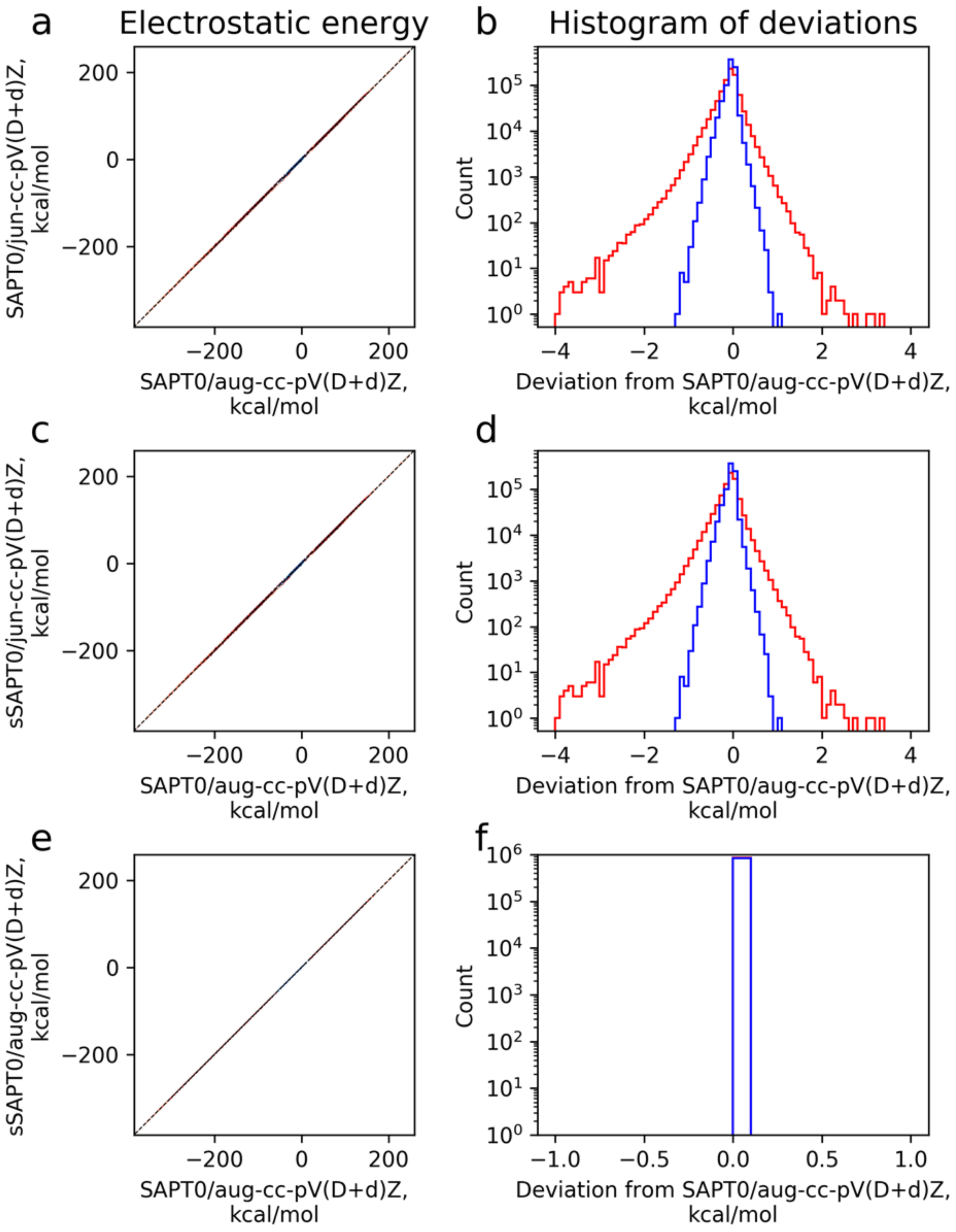

Supplementary Fig. S9: Exchange-repulsion component of SAPT0 energies calculated with different basis sets and exchange-scaling treatment compared to SAPT0/aug-cc-pV(D+d)Z. All panels are analogous to those in Supplementary Fig. S7, except the energies here are just the exchange-repulsion component of the SAPT0 interaction energies. Bin widths are 2 kcal/mol in both dimensions for 2D histograms (a, c, e) and 0.1 kcal/mol for 1D histograms (b, d, f). Note that the exchange-scaling algorithm does not modify this component, so the scaled and unscaled SAPT0/aug-cc-pV(D+d)Z energies are in perfect agreement (e, f).

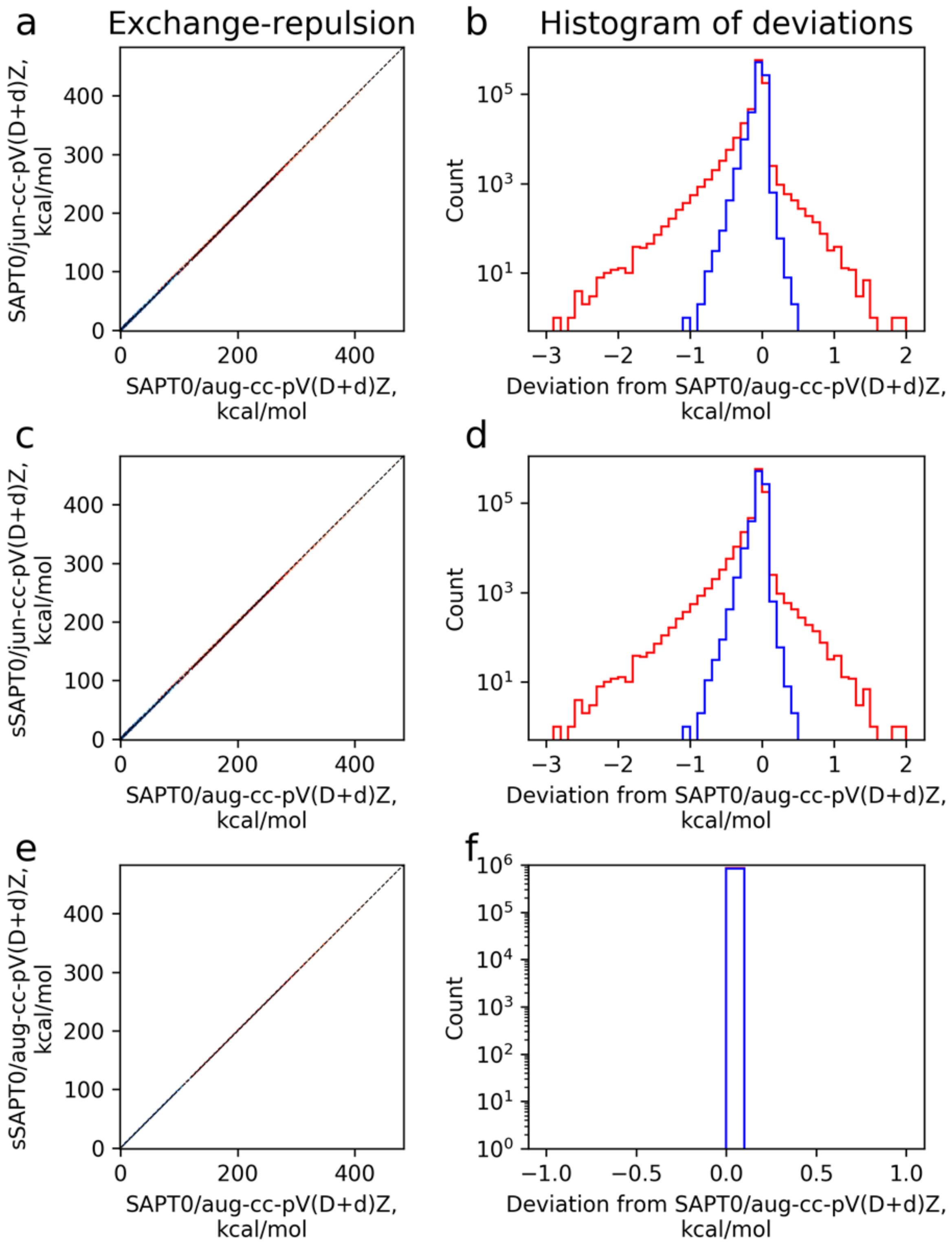

Supplementary Fig. S10: Induction/polarization component of SAPT0 energies calculated with different basis sets and exchange-scaling treatment compared to SAPT0/aug-cc-pV(D+d)Z. All panels are analogous to those in Supplementary Fig. S7, except the energies here are just the induction/polarization component of the SAPT0 interaction energies. Bin widths are 0.5 kcal/mol in both dimensions for 2D histograms (a, c, e) and 0.1 (b) or 1 kcal/mol (d, f) for 1D histograms. Extremely large deviations due to scaling sometimes occur for dimers with very low intermolecular distances (see also Supplementary Fig. S12).

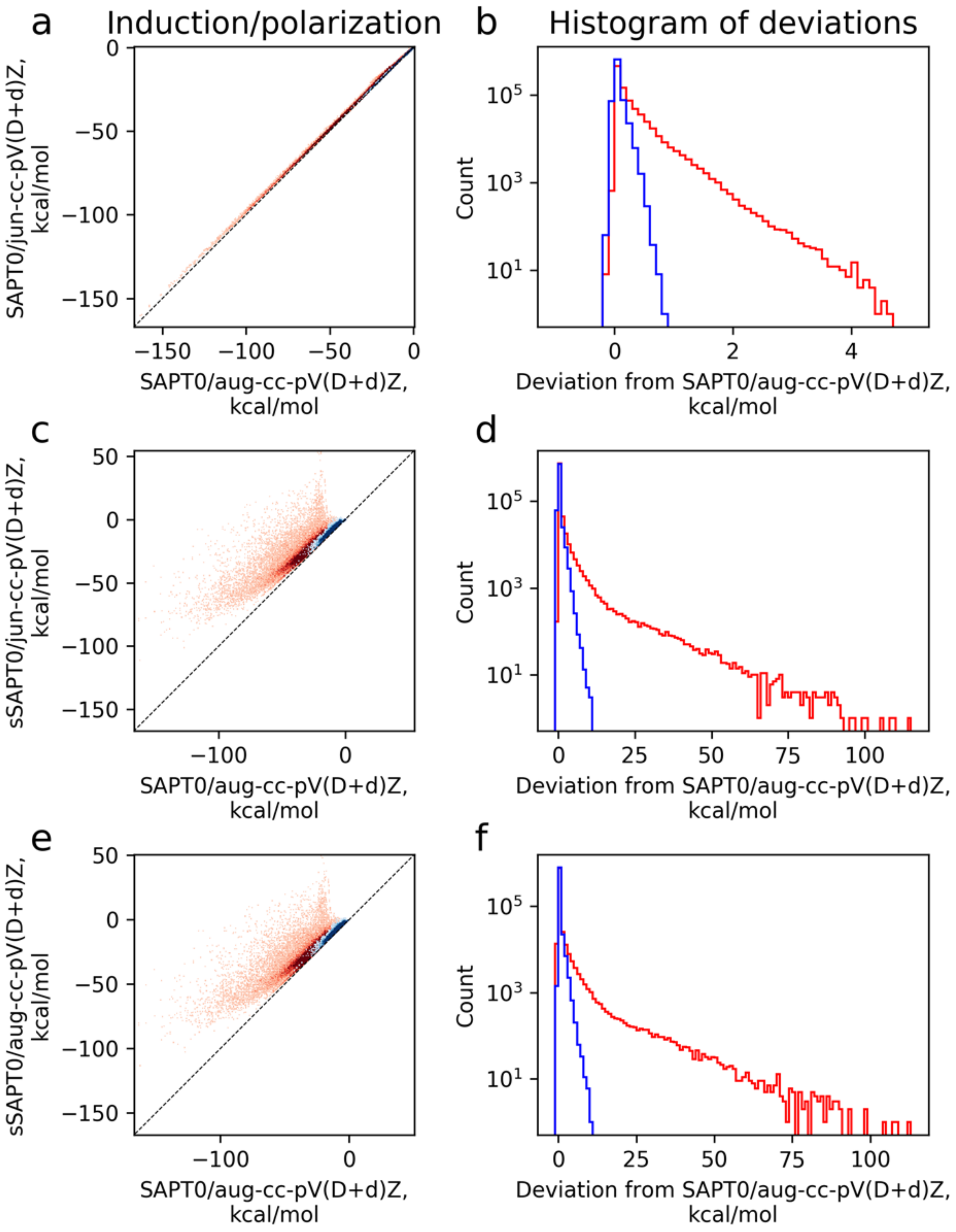

Supplementary Fig. S11: London dispersion component of SAPT0 energies calculated with different basis sets and exchange-scaling treatment compared to SAPT0/aug-cc-pV(D+d)Z. All panels are analogous to those in Supplementary Fig. S7, except the energies here are just the London dispersion component of the SAPT0 interaction energies. Bin widths are 0.1 kcal/mol in both dimensions for 2D histograms (a, c, e) and 0.1 kcal/mol for 1D histograms (b, d, f).

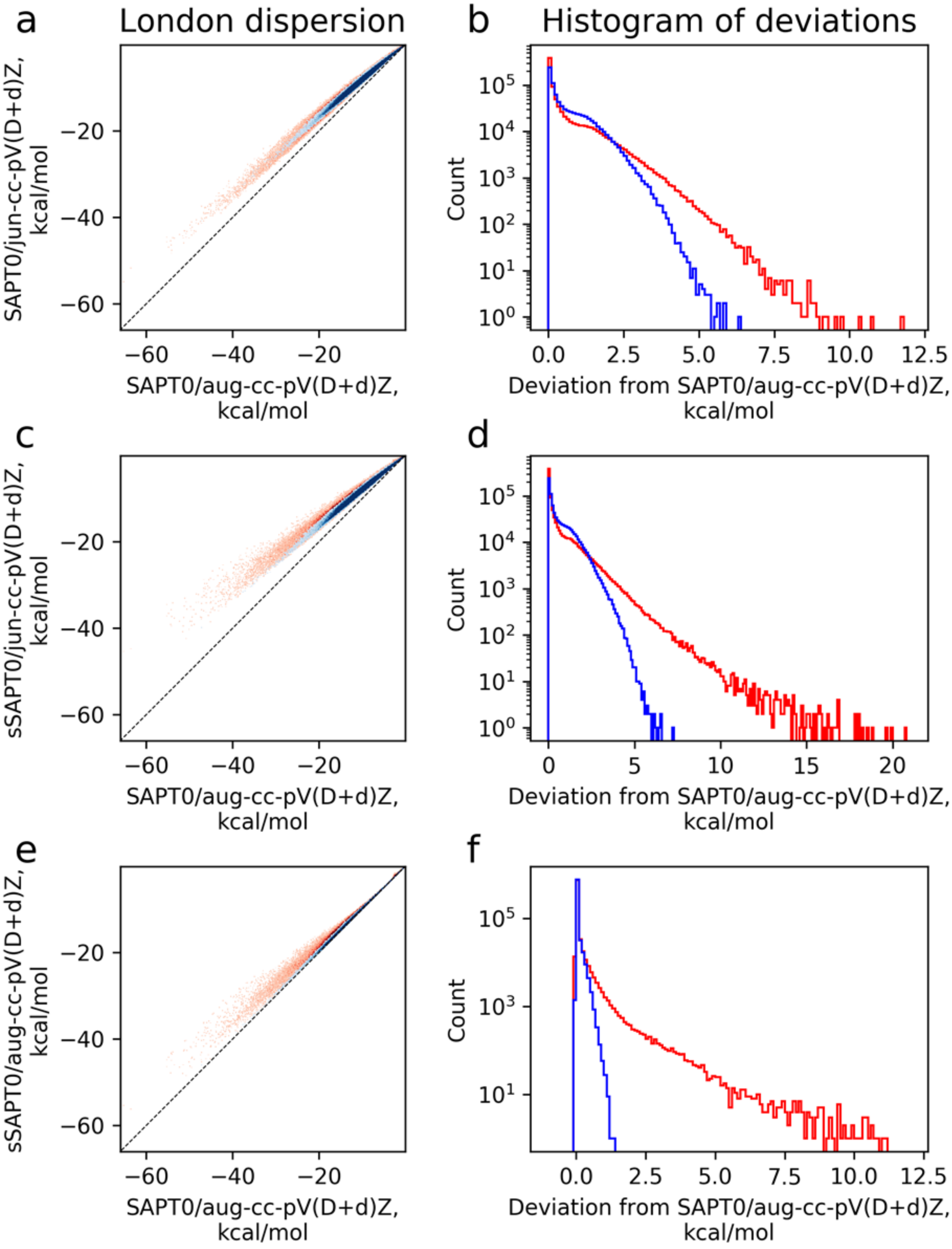

Supplementary Fig. S12: Dependence on r of the difference in the induction/polarization component of SAPT0 energies with and without exchange-scaling treament. Large values are observed for many complexes with low r, with both the jun-cc-pV(D+d)Z (a) and aug-cc-pV(D+d)Z basis sets (b). Blue dots represent dimers of two neutral monomers; red represent dimers with at least one charged monomer.

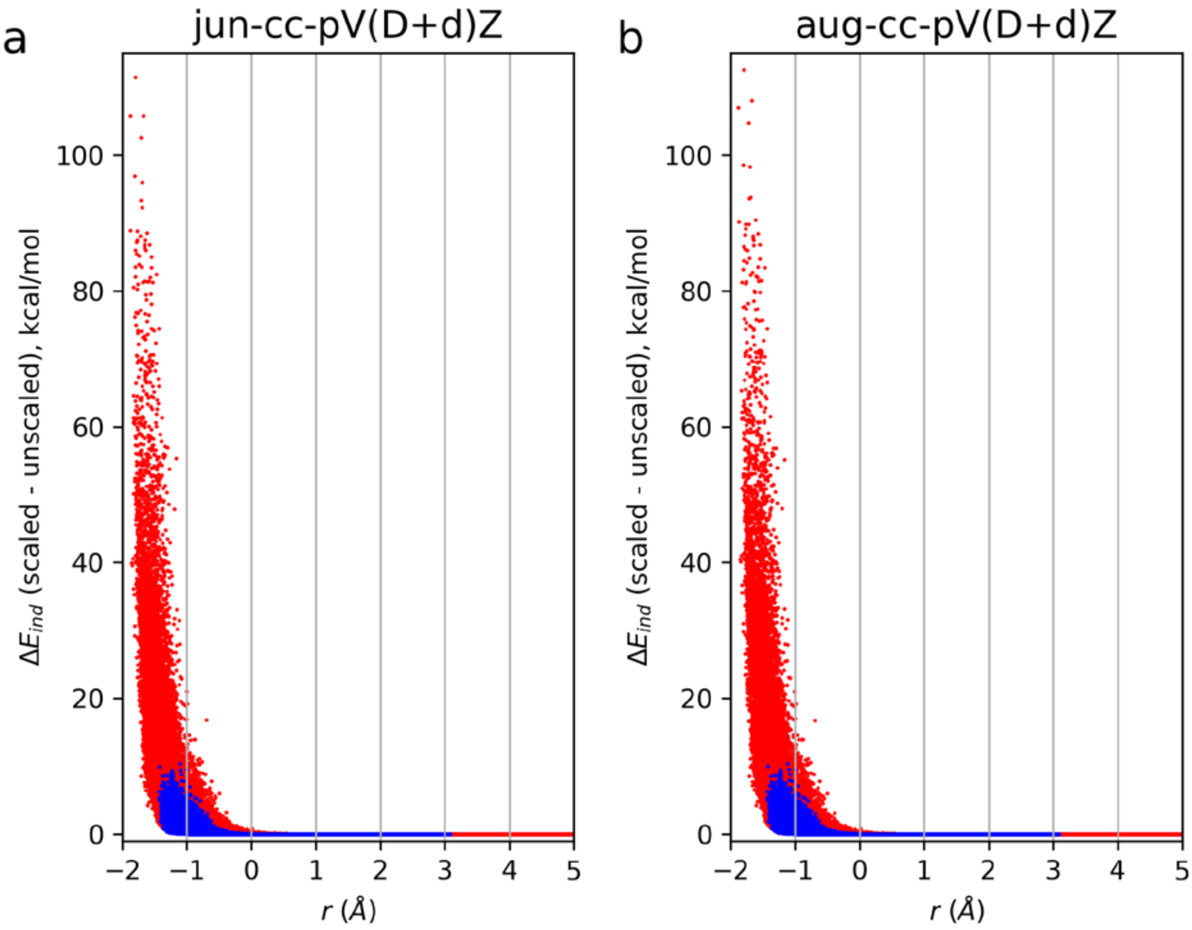

Supplementary Table S6: Interaction energies for representative dimers with close contacts. The four methods in the Splinter dataset are reported as well as counterpose-corrected CCSD(T)/CBS(aug-cc-v[TQ]Z; δ:aug-cc-pVDZ) are reported. Errors relative to CCSD(T)/CBS are noted in parentheses. Units are kcal/mol.

System name	CCSD(T)/CBS	SAPT0/ aug-cc-pV(D+d)Z	sSAPT0/ aug-cc-pV(D+d)Z	SAPT0/ jun-cc-pV(D+d)Z	sSAPT0/ jun-cc-pV(D+d)Z
CNCxNXdyNH2pY_pH-Naliph4_CCxCXCxdOXyOmY_macc-O1_5_ -1.72_161_33_112_-114_-161.xyz	-59.175 (0.000)	-79.895 (-20.720)	-24.385 (34.790)	-75.072 (-15.897)	-20.221 (38.954)
CCyNH3pY_pH-Naliph2_yOmYc1ccccc1_macc-O_18_ -1.70_168_104_108_164_37.xyz	-57.495 (0.000)	-80.545 (-23.050)	-24.581 (32.914)	-76.851 (-19.356)	-21.437 (36.058)
CCCxdOXyOmY-2_macc-O_c1ccynHpYcc1_pH-Narom_47_ -1.66_111_-77_138_80_47.xyz	-52.065 (0.000)	-66.738 (-14.673)	-11.675 (40.390)	-62.984 (-10.919)	-8.398 (43.667)
CCCxdOXyOmY-1_macc-O2_C1CyNH2pYC1_pH-Naliph2_15_ -1.73_162_-139_136_-179_113.xyz	-50.677 (0.000)	-70.370 (-19.693)	-10.787 (39.890)	-67.023 (-16.346)	-7.997 (42.680)
CCCxdOXyOmY-2_mgeneral_c1ccynHpYcc1_pgeneral_4_ -1.58_95_-113_80_-119_-48.xyz	-49.670 (0.000)	-55.152 (-5.482)	10.379 (60.049)	-48.953 (0.717)	15.652 (65.322)
CNCxNXdyNH2pY_pH-Naliph1_CCxdOXNyOmY_macc-O_36_ -1.74_155_-173_170_166_-20.xyz	-42.873 (0.000)	-63.224 (-20.351)	-9.333 (33.540)	-59.854 (-16.981)	-7.003 (35.870)
CCyNH3pY_pgeneral_OdCxyOmYXC1CC1_mgeneral_25_ -1.67_108_83_163_160_-35.xyz	-39.412 (0.000)	-57.822 (-18.410)	13.150 (52.562)	-53.211 (-13.799)	16.971 (56.383)
CCCxdOXyOmY-1_macc-O2_CyNHpY1CCCC1_pH-Naliph_3_ -1.69_136_-36_131_-23_-56.xyz	-34.971 (0.000)	-52.855 (-17.884)	3.645 (38.616)	-47.062 (-12.091)	8.870 (43.841)
CCCxdOXyOmY-1_macc-O1_C1CyNH2pYC1_pH-Naliph2_37_ -1.65_98_3_114_-175_-35.xyz	-32.480 (0.000)	-43.500 (-11.020)	14.436 (46.916)	-39.492 (-7.012)	17.691 (50.171)
CNCxNXdyNH2pY_pH-Naliph4_OdCxyOmYXC1CC1_macc-O1_12_ -1.72_135_63_131_89_144.xyz	-26.555 (0.000)	-41.031 (-14.476)	16.553 (43.108)	-37.306 (-10.751)	19.642 (46.197)
